# Patterns of rapid diversification in heteroploid *Knautia* sect. *Trichera* (Caprifoliaceae, Dipsacoideae), one of the most intricate taxa of the European flora

**DOI:** 10.1186/s12862-016-0773-2

**Published:** 2016-10-10

**Authors:** Božo Frajman, Ivana Rešetnik, Marjan Niketić, Friedrich Ehrendorfer, Peter Schönswetter

**Affiliations:** 1 Institute of Botany, University of Innsbruck, Sternwartestraße 15, A-6020 Innsbruck, Austria; 2 Faculty of Science, University of Zagreb, Marulićev trg 20, HR-10000 Zagreb, Croatia; 3 Natural History Museum, Njegoševa 51, 11000 Belgrade, Serbia; 4 Department of Botany and Biodiversity Research, University of Vienna, Rennweg 14, A-1030 Vienna, Austria

**Keywords:** Estimation of divergence times, Genetic versus traditional species groups, Haplotype sharing, Hybridisation, *Knautia*, Polyploidy, Random walk analysis

## Abstract

**Background:**

Polyploidy is one of the most important evolutionary pathways in flowering plants and has significantly contributed to their diversification and radiation. Due to the prevalence of reticulate evolution spanning three ploidy levels, *Knautia* is considered one of the taxonomically most intricate groups in the European flora. On the basis of ITS and plastid DNA sequences as well as AFLP fingerprints obtained from 381 populations of almost all species of the genus we asked the following questions. (1) Where and when did the initial diversification in *Knautia* take place, and how did it proceed further? (2) Did *Knautia* undergo a similarly recent (Pliocene/Pleistocene) rapid radiation as other genera with similar ecology and overlapping distribution? (3) Did polyploids evolve within the previously recognised diploid groups or rather from hybridisation between groups?

**Results:**

The diversification of *Knautia* was centred in the Eastern Mediterranean. According to our genetic data, the genus originated in the Early Miocene and started to diversify in the Middle Miocene, whereas the onset of radiation of sect. *Trichera* was in central parts of the Balkan Peninsula, roughly 4 Ma. Extensive spread out of the Balkans started in the Pleistocene about 1.5 Ma. Diversification of sect. *Trichera* was strongly fostered by polyploidisation, which occurred independently many times. Tetraploids are observed in almost all evolutionary lineages whereas hexaploids are rarer and restricted to a few phylogenetic groups. Whether polyploids originated via autopolyploidy or allopolyploidy is unclear due to the weak genetic separation among species. In spite of the complexity of sect. *Trichera*, we present nine AFLP-characterised informal species groups, which coincide only partly with former traditional groups.

**Conclusions:**

*Knautia* sect. *Trichera* is a prime example for rapid diversification, mostly taking place during Pliocene and Pleistocene. Numerous cycles of habitat fragmentation and subsequent reconnections likely promoted hybridisation and polyploidisation. Extensive haplotype sharing and unresolved phylogenetic relationships suggest that these processes occurred rapidly and extensively. Thus, the dynamic polyploid evolution, the lack of crossing barriers within ploidy levels supported by conserved floral morphology, the highly variable leaf morphology and unstable indumentum composition prevent establishing a well-founded taxonomic framework.

**Electronic supplementary material:**

The online version of this article (doi:10.1186/s12862-016-0773-2) contains supplementary material, which is available to authorized users.

## Background

Polyploidy is one of the most important evolutionary pathways in flowering plants and has significantly contributed to their diversification and radiation [1–4], for instance as the most frequent mode of sympatric speciation [5]. Polyploid lineages often exhibit complex relationships among each other as well as with their lower-ploid ancestors (e.g., [6–8]). This is fostered by the prevalence of gene flow from lower to higher ploidy levels, whereas the opposite case is considered rare [9, 10]. Multiple and recurrent formation of polyploids, (epi)genetic, transcriptomic and genomic changes as well as morphological, geographic and ecological divergence following polyploidisation are considered significant processes in the evolution of polyploids [11–17], and obviously increase taxonomic complexity. On the other hand, polyploids have lower speciation and higher extinction rates than diploids [18]; therefore, the high frequency of polyploids was suggested to be a consequence of their high formation rate rather than of accelerated diversification [19]. From the taxonomic viewpoint, polyploidy, or more generally reticulate evolution, thus clearly falls into the category “taxonomist’s nightmare—evolutionist’s delight” [20].

Due to the prevalence of reticulate evolution spanning three ploidy levels, *Knautia* L. (Caprifoliaceae, Dipsacoideae) is considered one of the taxonomically most intricate genera in the European flora [21, 22]. Dependent on taxonomic concepts it comprises about 50–55 species distributed in western Eurasia and northwestern-most Africa and is characterised by a lipid-rich elaiosome at the basis of the fruits [23]. Its traditional division into three sections was recently supported by molecular phylogenetic analyses of diploid species [24]. Whereas the species-poor, apparently early diverging annual sections *Knautia* (*x* = 8; *K. degenii*, *K. orientalis*) and *Tricheroides* (*x* = 10; *K. byzantina*, *K. integrifolia*) are centred in the eastern Mediterranean and comprise only diploids, the species-rich, mainly perennial section *Trichera* has its maximum diversity in Southern Europe and includes di-, tetra- and hexaploids based on *x* = 10. Extensive exploration of genome size and ploidy level variation in 381 populations of 54 species of sect. *Trichera* [25] has shown that di- and tetraploids are distributed across most of the distribution area of *Knautia*, whereas hexaploids are limited to the Balkan and Iberian Peninsulas and the Alps. Monoploid genome size varies considerably within the ploidy levels, but also within some of the species, and increases significantly towards the limits of the genus’ distribution.

The mostly perennial sect. *Trichera* possibly evolved from an annual ancestor [24]. Transition from an annual to a perennial life cycle was recently reconstructed also for *Delphinium* and *Lupinus* [26, 27], but stands in marked contrast with previous hypotheses [9, 21] suggesting life history evolution to proceed in the opposite way. Shallow diversification within sect. *Trichera* as well as extremely wide distribution of plastid haplotypes and—to a lesser extent—of ITS ribotypes, spanning almost the entire distribution of the genus, was considered indicative for rapid radiation and recent range expansion. In addition, extensive sharing of plastid haplotypes and ITS ribotypes across taxa indicates recurrent gene flow across species boundaries. Radiation in *Knautia* was suggested to have taken place 45–4.28 Ma [28], whereas diversification in the heteroploid *Centaurea* sect. *Acrocentron* (Asteraceae), *Dianthus* (Caryophyllaceae), *Scorzonera* (Asteraceae) and *Tragopogon* (Asteraceae) took place significantly later and has been associated with the onset of climatic and topographic changes in the Mediterranean region during the Pliocene and Pleistocene [28, 29].

In spite of the restriction to diploid cytotypes Rešetnik et al. [24] have shown that the shallow phylogenetic structure within *Knautia* prevents establishing a formal taxonomic framework; instead informal, genetically defined species groups were suggested. Some traditionally recognised groups (e.g., *K. dinarica*, *K. drymeia* and *K. montana* groups *sensu* Ehrendorfer [21, 30]) could be maintained, whereas several others (*K. arvensis*, *K. dalmatica*, *K. fleischmannii*, *K. longifolia* and *K. velutina* groups) were clearly polyphyletic and their diploid members were rearranged into the Xerophytic, Carinthiaca, Midzorensis, North Arvensis, South Arvensis, Pancicii and SW European Groups. Most of the traditional groups also include polyploid cytotypes of some heteroploid species as well as exclusively polyploid taxa. Additionally, Ehrendorfer [21, 30] recognised some entirely polyploid groups, such as *K. fleischmannii*, *K. subcanescens*-*K. persicina*, *K. dipsacifolia* (under the synonyme of *K. silvatica*) and *K. sarajevensis* groups, but diploids were recently discovered within some of them [24, 25].

One of the major problems in assessing the evolutionary history of heteroploid genera is the reticulate nature of polyploid speciation processes. In order to reconstruct these processes, the present study is based on three molecular markers, i.e. maternally inherited plastid DNA sequences as well as biparentally inherited nuclear ribosomal internal transcribed spacer (nrITS, in the following for simplicity termed ITS) sequences and amplified fragment length polymorphisms (AFLPs). The last method assesses genetic variation at a large number of anonymous loci mostly from the nuclear genome [31, 32] and was extensively applied to polyploid complexes (e.g., in *Hypochaeris*, [33]; *Rosa*, [34]; *Veronica*, [35]; *Leucanthemum*, [36]). The main objective of the present study is to elucidate the evolutionary relationships within the intricate sect. *Trichera* in which diploid and polyploid taxa were suggested to intermingle forming several tightly knit species groups [21, 30]. We asked the following questions. (1) Where and when did the initial diversification in *Knautia* take place, and how did it proceed further? (2) Did *Knautia* undergo a similarly recent (Pliocene/Pleistocene), rapid radiation as other genera with similar ecology and overlapping distribution such as *Dianthus* and *Tragopogon* [28, 29]? (3) Did polyploids evolve within the previously recognised diploid groups or rather from hybridisation between groups, and did polyploids form more extensively in certain diploid groups than in others? Finally, (4) dependent on the results we either assign the polyploid accessions to the informal species groups previously identified for diploids or propose additional species groups.

## Methods

### Plant material

Our sampling aimed at taxonomic completeness and inclusion of several populations, at least for the relatively widespread species. The final selection of samples was based on a previous exploration of ploidy level variation in 381 populations of 54 species [25] in order to represent each taxon with all its ploidy levels. As we were not interested in intra-population genetic diversity, we maximised the number of populations at the expense of the number of samples per population. Taxonomy follows Flora Europaea [37] with the exception of *K. csikii* not mentioned in Flora Europaea [38], the recently described *K. slovaca* [39], *K. serpentinicola* and *K. pseudolongifolia* [40] as well as the Iberian [41] and Turkish taxa [42], for which we followed newer or geographically more comprehensive treatments. In contrast to Frajman et al. [25] we include *K. wagneri* in *K. midzorensis*. Herbarium vouchers were revised by F. Ehrendorfer, a taxonomic expert of the group. Five populations could not be assigned to a species, i.e. four hexaploid populations from Velebit in Croatia (*K*. sp. 1: K102, K103, K105, K500) and one tetraploid population from Serbia (*K*. sp. 2: K218).

**Fig. 1:**
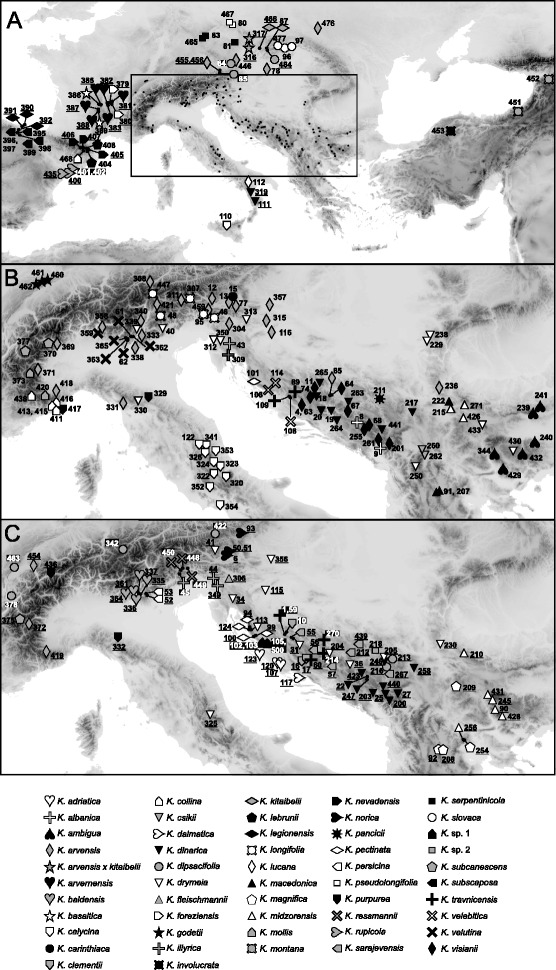
Sampled populations of 51 species of *Knautia* sect. *Trichera*. Population identifiers, which correspond to Additional file 1: Table S1, are underlined for tetraploid populations and white with black shading for hexaploid populations. Diploid populations are not highlighted. **a** distribution of diploid and polyploid taxa outside of the area enlarged in B and C; **b** distribution of diploid taxa modified from Rešetnik et al. [24]; **c** distribution of polyploid taxa. Taxa of the exclusively diploid sections *Knautia* and *Tricheroides* are not shown

Leaf material of one to five individuals per population and one to 35 populations per species (i.e., roughly proportional to the size of the species’ distribution areas; Fig. 1) was collected and immediately stored in silica gel; geographic coordinates were recorded for each population with a GPS. We aimed at sampling morphologically and ecologically homogenous populations and avoided possibly hybridogenous individuals. Voucher specimens are either deposited at the Institute of Botany, University of Innsbruck, Austria (IB), the Faculty of Science, University of Zagreb, Croatia (ZA), the Faculty of Agriculture, University of Zagreb, Croatia (ZAGR), the Faculty of Biology, University of Belgrade, Serbia (BEOU) or the Natural History Museum Belgrade, Serbia (BEO). Voucher numbers and collecting details are given in Additional file 1: Table S1; further information can be retrieved from the publicly accessible database of the BalkBioDiv project at http://www.uibk.ac.at/botany/balkbiodiv/?Sampling_sites.

### Molecular methods

Total genomic DNA was extracted from similar amounts of dried tissue (ca. 10 mg) with the DNeasy 96 plant mini kit (Qiagen, Hilden, Germany) following the manufacturer’s protocol.

Sequencing of the plastid *pet*N(*ycf*6)-*psb*M region and the nuclear ribosomal ITS region was performed as described by Rešetnik et al. [24], using the primers ycf6-F and psbM-R [43] and 17SE and 26SE [44], respectively. AFLP fingerprinting was performed for 258 populations of sect. *Trichera* and *K. integrifolia* with one to five individuals per population, using three primer combinations for the selective PCR (fluorescent dye in brackets): *Eco*RI (6-FAM)-ACA / *Mse*I-CTG, *Eco*RI (VIC)-ACG / *Mse*I-CTA, and *Eco*RI (NED)-ACC / *Mse*I-CTC. The AFLP laboratory procedure as well as the scoring approach followed Rešetnik et al. [24]; electropherograms were analysed with Peak Scanner version 1.0 (Applied Biosystems), and automated binning and scoring was performed using RawGeno version 2.0 [45], a package for the software R [46].

The error rate [47] was calculated as the ratio of mismatches (scoring of 0 vs. 1) over phenotypic comparisons in AFLP profiles of 43 replicated individuals. Non-reproducible fragments and fragments present in only one individual were removed from the dataset.

### Data analysis

#### Sequence data

Sequences were edited and manually aligned using Geneious Pro 5.3.6 [48]. All sequences were deposited in GenBank. Phylogenetic relationships in ITS and plastid data sets were inferred from maximum parsimony and Bayesian analyses. Maximum parsimony (MP) as well as MP bootstrap (MPB) analyses of both data sets were performed using PAUP 4.0b10 [49]. The most parsimonious trees were searched heuristically with TBR swapping, MulTrees off, and 100,000 replicates of random sequence addition for the ITS dataset and with 1000 replicates and swapping performed on a maximum of 1000 trees (nchuck = 1000) for the plastid dataset. All characters were equally weighted and unordered. The data set was bootstrapped using full heuristics, 1000 replicates, TBR branch swapping, MulTrees option off, and random addition sequence with five replicates. *Bassecoia hookeri* was used to root the trees and additional outgroup taxa were included, based on previous studies [23, 24].

Bayesian analyses were performed with MrBayes 3.2.1 [50] applying the substitution models proposed by the Akaike information criterion implemented in MrAIC.pl 1.4 [51]. Values for all parameters, such as the shape of the gamma distribution, were estimated during the analyses. The settings for the Metropolis-coupled Markov chain Monte Carlo (MC^3^) process included four runs with four chains each (three heated ones using the default heating scheme), run simultaneously for 10,000,000 generations each, sampling trees every 1,000th generation using default priors. The PP of the phylogeny and its branches were determined from the combined set of trees, discarding the first 1001 trees of each run as burn-in.

We constructed an ITS NeighbourNet network [52] of sect. *Trichera* using SplitsTree 4.12 [53, 54] to display possible conflicts in the data. We applied the Uncorrected_P method to compute the proportion of positions at which two sequences differ. Ambiguous base codes were treated as missing states. Plastid data were analysed using statistical parsimony as implemented in TCS 1.21 [55] with the connection limit set to 95 %; gaps were treated as fifth character state. For this analysis, indels longer than 1 bp were reduced to single base pair columns allowing those structural mutations to be counted as single base pair mutations only.

Divergence times were estimated using BEAST ver. 1.8.2 [56] on concatenated ITS and plastid datasets, using fossil calibration within the closely related Valerianaceae as described by Carlson et al. [57]. The dataset of *Knautia* was pruned to 27 accessions, of which 18 belong to sect. *Trichera* and were sampled from all major clades resolved in preliminary analyses of the complete datasets. In addition, several outgroup taxa were added. The calibration points were set as described by Carlson et al. [57] using lognormal prior distribution with mean = 4, standard deviation (SD) = 1 and offset 45 in the case of the crown group of Valerianaceae and with mean = 2, SD = 1 and offset 15 in the case of the crown group of *Valeriana*. The analyses were performed with a Birth-Death speciation prior, GTR + Γ substitution model parameters and an uncorrelated relaxed lognormal clock [58]. Two independent MCMC chains were run for 50,000,000 generations with tree and parameter values saved every 2,000th generation. Tracer 1.6.0 [59] was used to determine the degree of mixing, the shape of the probability density distributions, and 95 % credibility intervals for estimated divergence dates. Both the effective sample sizes and mixing were appropriate. FigTree 1.4.2 [60] was used to display the maximum clade credibility tree after combining the tree files using LogCombiner and summarising the information using TreeAnnotator (both programs available in BEAST package).

A continuous phylogeographic analysis using relaxed random walks [61] was performed on the combined sequence dataset including only *Knautia* (with exception of *K*. cf. *degenii* K272 exhibiting strongly conflicting positions in plastid and ITS trees; [24]) using BEAST v1.8.2 [62]. Substitution models proposed by MrAIC.pl 1.4 ([51]; HKY + Γ for ITS and GTR + Γ for plastid dataset) with estimated base frequencies were used for phylogeny inference and the trees were linked. A lognormal relaxed clock with a weakly informative prior on the clock rate (exponential with mean 0.001) was applied and a Bayesian skyline coalescent prior [63] with piecewise-linear skyline model was set. The diffusion process was modelled by a lognormal relaxed random walk process. We specified a prior exponential distribution on the standard deviation (SD) of the lognormal distribution with a mean of 5. Geographic coordinates recorded in the field with GPS were used as locality points for each population. We added random jitter with a window size 1.0 to the tips, as more individuals were sampled from the same location. The prior age of the root was set to 15.88 Ma with a normally distributed standard deviation of 4.5, which corresponds to the median age and 95 % highest posterior densities (HPD) interval of the corresponding node obtained from the dating analysis of the complete dataset. The analysis of the diffusion inference was run for 300 million generations, logging parameters every 10,000 generations. The performance of the analysis was checked in Tracer 1.6.0 [59], which was also used to construct a lineage-through-time (LTT) plot from the combined posterior distribution of sampled tree topologies, in order to display the diversification rate dynamics in the evolutionary history of *Knautia*. The maximum clade credibility tree (MCC) was produced and annotated by Tree Annotator (part of the BEAST package) after removing burnin and visualised with FigTree 1.4.2 [60]. The diffused MCC tree with annotated diffusion estimates was visualised in SPREAD v.1.0.6 [64] and projected together with polygons representing ancestral areas on a geo-referenced map using ArcGIS 10.3.

Diversification rates were estimated using Magallón and Sanderson’s whole-clade method [65], which does not assume complete taxon sampling. Rates were calculated for both crown and stem groups, at two extremes of the relative extinction rate (ε = 0, no extinction; and ε = 0.9, high rate of extinction; extinction rate being expressed as a fraction of the speciation rate), as implemented in the R package GEIGER [66].

#### AFLP data

**Fig. 2:**
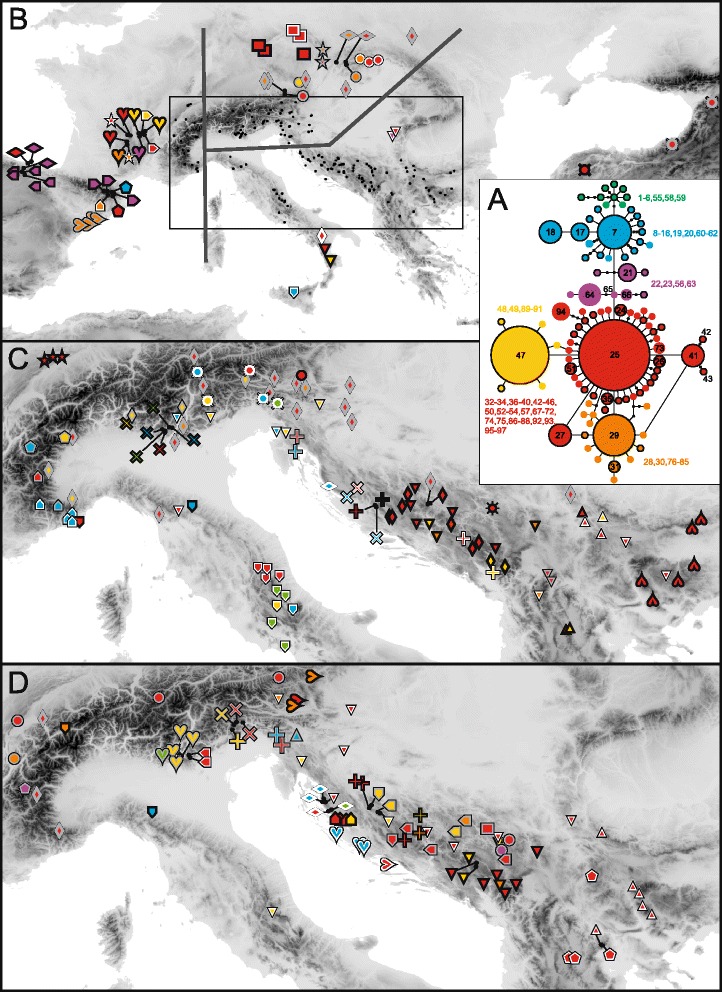
Plastid DNA variation in populations of 51 species of *Knautia* sect. *Trichera* based on *pet*N(*ycf*6)-*psb*M sequences. **a** statistical parsimony network of the 97 plastid haplotypes encountered; numbering corresponds to Additional file 1: Table S1 (the numbers 1–57 correspond to diploid accessions from Rešetnik et al. [24]); the size of the circles is proportional to the square-root transformed frequency of the respective haplotype; haplotypes not sampled are shown as small black dots. Only haplotypes retrieved from at least two individuals as well as those mentioned in the text are labelled separately. Haplotypes present in diploids are identified by a black outline. **b**–**d** geographic distribution of haplotypes. Symbols for the species are as in Fig. 1; their colour filling corresponds to the haplotype groups shown in **a**. The grey lines in **b** delimit three areas for which separate NeighbourNets of AFLP relationships complemented with plastid haplotypes are given in Figures S7–S9 within the Additional file 8

A Neighbor-joining (NJ) analysis based on a matrix of Nei-Li distances [67] and rooted with *K. integrifolia* from section *Tricheroides* was conducted and bootstrapped (1000 pseudo-replicates) with TREECON 1.3b [68]. A non-model-based approach, nonhierarchical K-means clustering [69] was chosen because of the presence of three ploidy levels, and performed using a script of Arrigo et al. [70] in R. This approach has recently been successfully applied in the analysis of genetic structure of AFLP datasets in polyploid complexes [70–72]. We performed 50,000 independent runs (i.e., starting from random points) for each assumed value of K (i.e. the number of groups ranging from 2 to 20). The K-means clustering results were displayed on a NeighborNet diagram produced with SplitsTree 4.12 [54] from a matrix of uncorrected P-distances; *K. integrifolia* was not included. Splits with a weight < 0.001 were excluded to aid legibility. In order to simplify the interpretation of the data we also present NeighborNets of three geographical areas supplemented with bootstrap values (1000 pseudo-replicates); the circumscription of the regions is given in Fig. 2.

## Results

The number of terminals, included characters, number and percentage of parsimony informative characters, number and lengths of MP trees, consistency and retention indices for both DNA regions, as well as the model of evolution proposed by MrAIC and used in MrBayes analyses are presented in Table 1.

**Table 1:** Matrix and phylogenetic analysis statistics for ITS and the plastid marker *pet*N(*ycf*6)-*psb*M as well as substitution models proposed by MrAIC and used in the Bayesian analyses

	ITS	*pet*N(*ycf*6)-*psb*M
Number of terminals	278	274
Number of included characters	948	1444
Number / percentage of parsimony informative characters	192 / 20.3 %	103 / 7.1 %
Number of MP trees	17,635	52
Length of MP trees	539	244
Consistency index (CI; excluding uninformative characters)	0.646 (0.585)	0.881 (0.797)
Retention index (RI)	0.923	0.956
Substitution model	HKY + Γ	GTR + Γ

### Plastid sequence data

The *pet*N(*ycf*6)-*psb*M sequences of sect. *Trichera* were 1230 (K388 and K468) to 1282 bp (K246) long and the alignment was 1444 bp long. The relationships inferred among the sections of *Knautia* were congruent with our previous study [24]. Within sect. *Trichera* several clades with poorly resolved and insufficiently supported relationships were unravelled (Additional file 2: Figure S1). Tetraploids were found in all main clades, whereas hexaploids are more limited. The parsimony haplotype network (Fig. 2a; from here on, the term haplotype is restricted to plastid sequences) exhibited a simple structure: in total, 97 haplotypes were retrieved, of which haplotypes H1–H57 were also present in diploid individuals [24]. The most frequent haplotype H25 was found in 31.2 % of the samples. It connected to 39 closely related haplotypes differing in only one or two steps, whereas H42 and H43 (both derived from H41) were separated by three steps and H94 by four steps (Red Haplotype Group). The satellites of H25 separated by one step included also the second-most frequent haplotype H47 (Yellow Haplotype Group, 13.8 %), H29 (Orange Haplotype Group) and H65 (Violet Haplotype Group). Haplotype H47 had five satellites separated by one step, while H29 had 13 satellites separated by one to four steps, some of which connected also to haplotypes belonging to the Red Haplotype Group. The Violet Haplotype Group distributed in Iberia and southeastern Europe was genetically most heterogeneous. It was constituted by four haplotypes (including H64) derived from H65 and three haplotypes (including H21) connecting to a not sampled haplotype, to which also H7 was connected (Blue Haplotype Group). Haplotype H7 was surrounded by 16 haplotypes separated by maximally three steps. Finally, H1, giving rise to eight haplotypes separated by one to five steps, was connected to H7 by two steps (Green Haplotype Group).

The geographic distribution of the haplotype groups is illustrated in Fig. 2b–d. Individuals carrying haplotypes of the Red Haplotype Group were distributed throughout most of the sampling area from the Pyrenees to the Caucasus. The second-most widely distributed haplotype group (Orange Haplotype Group) ranged from the eastern Pyrenees over the Alps to the central Balkan Peninsula, but was absent from the Apennines. The Yellow Haplotype Group was widely distributed from the French Massif Central over the Alps to the Apennines and to the central Balkan Peninsula. The Green Haplotype Group was restricted to *K. calycina* from the Apennines, four accessions of *K. baldensis*, *K. longifolia* and *K. velutina* from the southern Alps and a single accession of *K. travnicensis* from the Balkans. The Violet Haplotype Group was mostly restricted to the Iberian Peninsula, with occurrences of single accessions in the Massif Central (*K. arvernensis*), the Western Alps (*K. subcanescens*), the Southern Carpathians (*K. drymeia*) and the central Balkan Peninsula (*K. dipsacifolia*).

### ITS

Raw ITS sequences of sect. *Trichera* were 849 (K229 and K250) to 858 bp (K089 and K109) long and the alignment was 948 bp long. Polymorphisms were detected in most sequences (Additional file 1: Table S1); among diploid accessions there were 68 samples (46 %) without polymorphism, whereas the highest number of detected polymorphisms was ten (in *K. midzorensis* K271), among tetraploid accessions 24 (25 %) of samples showed no polymorphisms and the highest number of polymorphisms was 17 (in *K. norica* K051), and among hexaploid accessions two (9.5 %) of samples showed no polymorphisms whereas the highest detected number was ten (in *K. travnicensis* K270). The relationships inferred among the sections of *Knautia* were congruent with our previous study [24]. Within sect. *Trichera* several clades with poorly resolved and insufficiently supported relationships were inferred (Additional file 3: Figure S2).

**Fig. 3:**
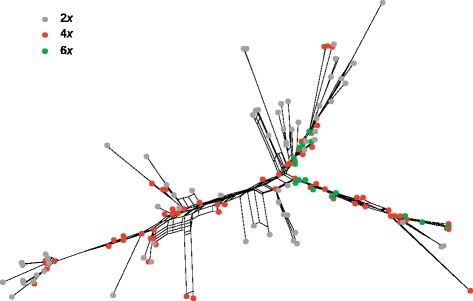
Internal Transcribed Spacer (ITS) variation in *Knautia* sect. *Trichera* illustrating dispersion of cytotypes over the network. Relationships are visualised as NeighbourNet diagram based on uncorrected P distances; a fully labelled version is presented in Additional file 4: Figure S3

The NeighbourNet network of ITS ribotypes (Additional file 4: Figure S3A) revealed a structure similar to that inferred from diploid accessions only ([24]; Additional file 4: Figure S3B). The main difference was that several accessions of *K. carinthiaca*, *K. dinarica*, *K. illyrica*, *K. longifolia*, *K. magnifica*, *K. midzorenzis*, *K. norica*, *K. pancicii*, *K*. sp. 2 as well as a few samples of *K. arvensis*, *K. arvernensis*, *K. csikii*, *K. dipsacifolia*, *K. drymeia* and *K. sarajevensis* were positioned along the split between the two main terminal groups identified from diploid accessions only [24]. One major group was genetically fairly homogenous and included most taxa of the South Arvensis Group, plus a few samples of *K. csikii*, *K. dinarica*, *K. drymeia*, *K. sarajevensis* and *K. slovaca*. The second major group comprised most other taxa (also including a few samples of *K. arvensis*, *K. dinarica* and *K. drymeia*) and was genetically highly diverse, with several strongly weighted splits. Many species exhibited unrelated ribotypes (Additional file 5: Figure S4). Whereas tetraploids were positioned all over the NeighbourNet, hexaploids were limited to two lineages (Fig. 3).

### Divergence time estimation, diversification dynamics and continuous phylogeographic analysis

**Fig. 4:**
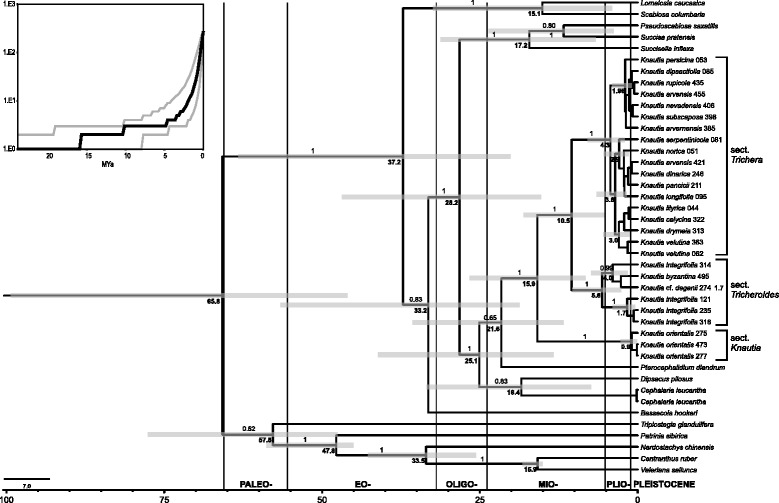
Bayesian consensus chronogram of the concatenated ITS and plastid datasets obtained with BEAST. Numbers above branches are PP values >0.50 (they were omitted within the crown groups of section *Trichera*), numbers in bold associated with nodes indicate the mean crown group age in millions of years of the clade diversifying at that node and the bars correspond to the 95 % highest posterior densities of the age estimates. Population identifiers correspond to Additional file 1: Table S1. The insert shows a lineage-through-time plot displaying the dynamics of diversification of *Knautia*. The black line represents the MCC tree from the BEAST analysis and the grey lines represent the interval resulting from all sampled trees after burnin

The overall phylogenetic relationships inferred by BEAST analysis of the concatenated ITS and plastid datasets including several outgroup taxa (Fig. 4) were congruent with previous studies [24, 57] and resulted in poor resolution within *Knautia* sect. *Trichera*. In addition, the inferred divergence times in the outgroup were slightly older, but largely within the ranges (95 % highest posterior densities, HPDs) inferred by Carlson et al. [57]. In our analysis the origin of *Knautia*, i.e. its divergence from *Pterocephalidium* was dated to the early Miocene 21.6 (11.7–35.7) Ma, whereas its diversification started in the mid Miocene at 15.9 (8.2–26.6) Ma with the divergence of sect. *Knautia*. The split of sections *Trichera* and *Tricheroides* might have occurred at 10.5 (5.3–18.0) Ma and the divergence within sect. *Trichera* in the Pliocene 4.3 (2.1–7.9) Ma, with the main phase of diversification dated to Pliocene and Pleistocene. The divergence dates obtained by the BEAST analysis for the dataset pruned to *Knautia* (not shown) were highly congruent with the analysis of the entire data set and the HPDs were strongly overlapping: the beginning of diversification of *Knautia* was dated to 16.2 (7.7–23.9) Ma, the split between sections *Trichera* and *Tricheroides* to 10.7 (3.9.–18.4) Ma and the onset of diversification in sect. *Trichera* to 4.0 (1.1–7.6) Ma.

**Fig. 5:**
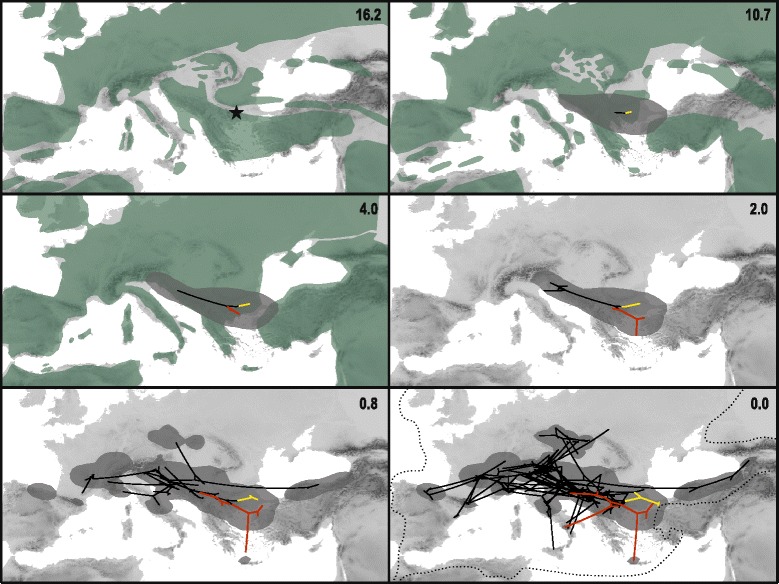
Snapshots of estimated ancestral node areas in the Maximum Clade Credibility tree (obtained with BEAST) of combined ITS and plastid datasets of *Knautia* at different time horizons as visualised using the software SPREAD. The starting point of diversification is indicated with an asterisk in the upper left figure, the 80 % highest posterior density areas for nodes are indicated as grey polygons, and the time scale of diversification is indicated in million years before present in the upper right corner in each panel. Distribution of land in the corresponding periods is indicated by green polygons in the two upper and the left middle panels (from Rögl [81]: Figs. 8 and 12 for the two upper panels, and from Meulenkamp and Sissingh [85]: Fig. 7 for the left middle panel). The coloured lines show the diversification of *Knautia* sections: black, section *Trichera*; yellow, section *Knautia*; red, section *Tricheroides*. The distribution of *K*. sect. *Trichera* is indicated by a dashed line in the right lower panel

The continuous phylogeographic analysis (Fig. 5) revealed that the beginning of diversification of *Knautia* was centred in the Eastern Mediterranean, roughly in the area of the eastern Balkan Peninsula, from where it slowly spread to the neighbouring regions. The diversification of sect. *Trichera* might have started in central parts of the Balkan Peninsula roughly 4 Ma, from where it significantly expanded its range only in the last 1.5 Ma. Accordingly, most extant lineages originated in the Plio- and Pleistocene, as displayed in the lineage-through-time (LTT) plot (Fig. 4). Based on the estimate of 4.0 Ma (1.1–7.6) for the onset of diversification of *K*. sect. *Trichera* and 50 species, we estimated the diversification rate to be approximately 0.42–2.87 species/Myr for the crown group, assuming no extinction, and 0.23–1.54 species/Myr, assuming a high proportion of extinction (e = 0.9). These, as well as stem-group-based diversification rates, are presented in Table 2.

**Table 2:** Diversification rates (birth–death) in species per Myr in *Knautia* sect. *Trichera* assuming 50 species, following the method of Magallón and Sanderson [65] and using 95 % highest posterior density intervals of the age estimates from BEAST analysis of a dataset pruned to *Knautia*. ε, extinction rate

Clade	Diversification rate
crown group, ε = 0	0.42–2.87
crown group, ε = 0.9	0.23–1.54
stem group, ε = 0	0.22–0.74
stem group, ε = 0.9	0.10–0.33

### AFLP data

Thirty-two individuals failed to produce reliable AFLP profiles and were excluded, resulting in a final dataset including 350 individuals. A total of 1334 AFLP fragments were scored; 174 bands were found in only one individual and were excluded from further analyses. The average replicate error rate (according to Bonin et al. [47]) was 2.17 %. Analyses of sect. *Trichera* (i.e., excluding the outgroup were based on a matrix with 345 individuals and 1149 AFLP fragments. We acknowledge that the number of fragments is high (on average, one fragment was scored every 1.2 bp), which could introduce considerable homoplasy. Empirical tests, however, showed that reducing the number of fragments by applying more conservative scoring strategies yielded considerably worse resolution in terms of tree structure and bootstrap support. This indicates that the increased amount of data was not outweighed by increased homoplasy.

**Fig. 6:**
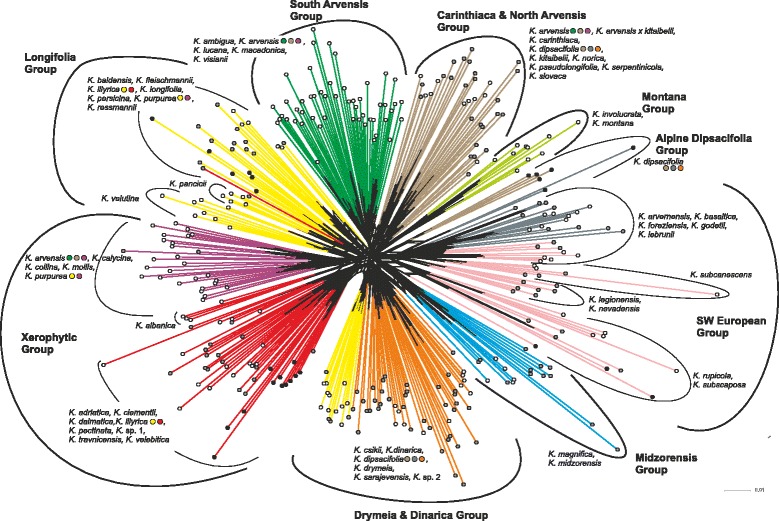
Amplified Fragment Length Polymorphism (AFLP) variation in 251 populations of 51 species of *Knautia* sect. *Trichera*. Relationships are visualised as NeighbourNet diagram based on uncorrected P distances. Dots at the tips of branches indicate ploidy levels: white, diploid; grey, tetraploid; black, hexaploid. The colours of individual branches indicate the ten genetic clusters identified as optimal solution by K-means clustering. Nine groups, whose circumscription was additionally informed by the clustering of diploid accessions [24] and the topology of the NeighbourNet, are indicated by thick black lines. Species assigned to more than one cluster are highlighted with dots, whose colours reflect all clusters a species is assigned to. An enlargeable version of Fig. 6 with labelling of terminal splits is presented as Additional file 7: Figure S6

The NJ analysis (Additional file 6: Figure S5) supports the divergence of sect. *Tricheroides* represented by *K. integrifolia* from sect. *Trichera* (bootstrap support, BS, 100). *Knautia pancicii* (BS 100) was sister to the remaining species with low support (BS 63). Species forming well-supported (BS ≥ 95) branches included *K. albanica*, *K. carinthiaca*, *K. collina*, *K. involucrata*, *K. lebrunii*, *K. mollis* and *K. subscaposa*. The backbone of the tree was unresolved. Nonhierarchical K-means clustering revealed an optimal separation of the dataset into ten groups that showed good overall congruence with the NeighbourNet diagram (Fig. 6; Additional file 7: Figure S6 presents the NeighbourNet diagram from Fig. 6 complemented with population IDs and species names). Most species groups previously identified in diploid accessions only [24] are still recognizable in the heteroploid data set (Fig. 6). The only supported species groups were the Midzorensis Group (BS 61) and the Montana Group (BS 99). In order to make reading easier and to contrast genetic relationships with taxon-specific average leaf shapes, separate NeighbourNet diagrams are shown for southwestern, central and southeastern Europe in the Additional file 8: Figures S7–S9.

## Discussion

### Spatiotemporal diversification of *Knautia* and radiation of *Knautia* sect. *Trichera*

*Knautia* is sister (Additional file 2: Figure S1 and Additional file 3: Figure S2; [24]) to the monotypic western Mediterranean *Pterocephalidium* [41], which constitutes the tribe Pterocephalidieae together with the also monotypic montane southeastern African half-shrub *Pterothamnus* [73]. The split between *Pterocephalidium* and *Knautia* likely occurred in the Early Miocene (Fig. 4), and the diversification of *Knautia* was centred in the Eastern Mediterranean as reconstructed by relaxed random walks (Fig. 5). The Mediterranean region is considered one of the Earth’s 25 biodiversity hot spots [74], hosting ca. 24,000 plant species of which 60 % are endemic [75]. Despite its younger age, the Eastern Mediterranean appears to be more diverse than the Western Mediterranean [76] and is thus often considered a reservoir for plant evolution or a cradle for lineage diversification [77–80]. This, obviously, was also the case in *Knautia*.

Applying the same calibration points and estimating similar divergence times within the outgroup as Carlson et al. [57] the dating analysis (Fig. 4) suggests that the onset of diversification of *Knautia* was in the Middle Miocene ca. 16 Ma (Fig. 4), when Mediterranean Sea and Paratethys transgressed and the Eastern Mediterranean was a mosaic of bigger and smaller islands [81]. Roughly 5 Ma later the divergence between sections *Trichera* and *Tricheroides* might have started in approximately the same region. At that time—the land-sea configuration was already similar to the present [81]—*Knautia* was distributed throughout the central and eastern Balkan Peninsula and westernmost Anatolia (Fig. 5b). The genus persisted in the same area for another 6 Ma, and the diversification of sect. *Trichera* started in central parts of the Balkan Peninsula roughly 4 Ma. Extensive spread of sect. *Trichera* out of the Balkans started in the Pleistocene about 1.5 Ma, extending the range westwards along the southern margins of the Alps and eastwards to central Anatolia. All other areas were colonised in the last 1 Ma. The species-poor sections *Knautia* and *Tricheroides* remained centred in the Eastern Mediterranean, only *K. integrifolia* from sect. *Tricheroides* spread to the Western Mediterranean (e.g., [41, 82]). These sections’ diversity increased to only two species each [24], whereas sect. *Trichera* underwent rapid diversification in the Pliocene, as displayed in the LTT plot (Fig. 4), and resulted in the ample geographic distribution of haplotypes and ribotype groups, which are shared across species boundaries and ploidy levels (Figs. 2 and 3).

Our reconstruction of massive Pliocene and Pleistocene radiation within sect. *Trichera* is in stark contrast with Bell et al. [28], who suggested that the radiation in *Knautia* resulting in today’s species diversity took place much earlier (45–4.28 Ma). Their estimate likely reflects the initial, sectional diversification of *Knautia*, whereas the radiation of *K*. sect. *Trichera* is certainly younger. The estimated diversification rate of the crown group of 0.42–2.87 species/Myr (assuming no extinction) overlaps with the rate estimated for *Tragopogon* (0.84–2.71 species/Myr; [28]), *Dianthus* (0.66–3.89 species/Myr; [29]) and some other European-centred genera with rapid rates of diversification (reviewed by Valente et al. [29]). Consequently, diversification of sect. *Trichera* took place at a similar time horizon as in other genera such as *Astragalus*, *Centaurea*, *Dianthus*, *Scabiosa Scorzonera* and *Tragopogon* [28]. As in these genera, it was likely triggered by climatic and topographic changes in the Mediterranean following the Messinian Salinity Crisis in the late Miocene [83–86], when the warm and humid climate of the Miocene shifted to clear seasonality with summer droughts and cold, humid winters [29]. Furthermore, uplift of the southern European mountain systems [85] led to an increased altitudinal differentiation in the vegetation [29, 87]. Subsequently, the climatic oscillations of the Pleistocene likely stimulated the alternation of phases of allopatric divergence with periods of secondary contacts of previously isolated lineages [88–90], thereby reshuffling species distributions and triggering reticulation and polyploidisation (see below). Finally, the spread of grasslands in the course of Holocene anthropogenic deforestation certainly contributed to the range expansion of the nowadays most widespread species, *K. arvensis*, and triggered secondary contacts with other, previously isolated lineages [24, 91].

### Polyploid *Knautia* mostly evolved within previously recognised diploid groups

Diversification of sect. *Trichera* was strongly enhanced by polyploidisation, which occurred independently many times, but did not significantly influence the overall genetic pattern inferred for diploids [24]. The inclusion of polyploids in the diploid AFLP framework revealed that with the exception of hexaploid *K. dipsacifolia* all tetraploids and hexaploids are nested within diploid groups (Fig. 6). Tetraploids are observed in almost all evolutionary lineages, whereas the much rarer hexaploids are restricted to a few AFLP groups (Fig. 6). The ITS data also support this pattern, as hexaploids appear only in two lineages revealed by the NeighborNet, whereas tetraploids are present in all lineages (Fig. 3). Hexaploids are also geographically more limited, with larger distribution areas in the Alps and north of them and with small isolated occurrences on the western Balkan Peninsula and the northeastern Iberian Peninsula (Fig. 1; [25, 30]). In contrast, tetraploids are present throughout the distributional range of the genus except for its extreme east [25].

Within the eleven AFLP groups previously inferred from diploid accessions [24], polyploids originated in all except for three: (1) the Pancicii Group comprising only *K. pancicii*, (2) the Montana Group constituted by *K. involucrata* and *K. montana*, two species from Anatolia and the Caucasus, the very East of the genus’ distribution area, and (3) the South Arvensis Group (Fig. 6). The last case is of particular interest, as this group is well covered by our sampling. Ecologically, members of the South Arvensis Group share a preference of dry grasslands with many species of the strongly heteroploid Xerophytic Group (Fig. 6) precluding inference of ecological causes for the absence of polyploidy in the South Arvensis Group. A possible explanation for the observed pattern might be the relative stability of environmental conditions in the areas south of the Alps throughout the Pleistocene [76, 92, 93] conferring distributional stasis at least on a larger scale [94]. Nevertheless, polyploidisation was extensive in these areas in other groups of sect. *Trichera*. The shallow genetic structure as well as the scattering of tetraploids throughout most of the phylogeny (Figs. 2 and 3, Additional file 2: Figure S1, Additional file 3: Figure S2) make it difficult to establish a minimal number of polyploidisation events giving rise to tetraploids in sect. *Trichera*, which appear in six AFLP groups. Hexaploids originated at least four times, i.e. in the heteroploid Longifolia, Xerophytic and SW European Groups as well as in the exclusively hexaploid Alpine Dipsacifolia Group (Fig. 6). Similarly, the parsimony network of plastid haplotypes suggests at least eight and three independent origins for haplotypes retrieved from tetraploids and hexaploids, respectively. We emphasise that these numbers should be viewed with caution and represent minimum estimates.

Some of the previously inferred species groups [24] were strongly inflated by the inclusion of polyploid accessions (Figs. 3 and 6; circumscription and detailed characterisations are provided in Table 3 and Additional file 8). *Knautia velutina* and a few accessions of *K. illyrica* and *K. purpurea* previously included in the Xerophytic Group had to be transferred to the Longifolia Group (Fig. 6). Whereas the Midzorensis and SW European Groups could be maintained, the previously recognised Carinthiaca Group and North Arvensis Group on the one hand and the Drymeia Group and the Dinarica Group on the other hand were not separable upon the inclusion of polyploids. Therefore, they are here united as Carinthiaca & North Arvensis Group and Drymeia & Dinarica Group (Fig. 6). As in our previous study [24] the widespread and morphologically heterogeneous [72] diploid-tetraploid *K. arvensis* is non-monophyletic and falls into three groups (Fig. 6). The same applies to the tetra-hexaploid *K. dipsacifolia*, which appears in three different groups, one of which is the newly proposed Alpine Dipsacifolia Group. Further cases of polyphyly concern populations of *K. illyrica* and *K. purpurea*, which occur in two different groups. Future research will show if these four species need to be split into several taxonomic entities. As we expected that separating the AFLP data set into three regional groups (southwestern, central and southeastern Europe; for a circumscription see Fig. 2b) would improve readability and resolution, we present regional NeighbourNets in the Additional file 8: Figures S7–S9. Whereas BS support values for species and a few species clusters tended to increase because of the overall reduced variability in the regional data sets, some species groups did not form clusters anymore (e.g., Carinthiaca & North Arvensis Group, South Arvensis Group; Additional file 8: Figure S8). Addition of typical leaf shapes drawn from specimens of investigated populations (Additional file 8: Figures S7–S9) shows that leaf shape, which is one of the most important characters in *Knautia* alongside indumentum composition and quantity [21, 37], varies strongly within most of the species groups. However, this is not surprising as some of the species exhibit highly divergent leaf shapes even within populations (e.g., *K. arvensis*, *K. dinarica*, *K. nevadensis*, *K. travnicensis*; P. Schönswetter & B. Frajman, field observations).

**Table 3:** Comparison of the species groups within *Knautia* sect. *Trichera* proposed by us with those of Ehrendorfer [21, 30]. Information in squared brackets refers to accessions of the same species belonging to different AFLP groups. The addition “p.p.” after a species’ name indicates that the species is included in more than one AFLP groups. Distributions of individual species are characterised based on floras as well as on the author’s field observations

AFLP groups and constituent species	Groups recognised by Ehrendorfer (1962a, 1981)	Ploidy levels of AFLP groups and constituent species	Habitat preference of individual species	Distribution type of AFLP groups and distribution of individual species	Morphological characteristics of AFLP groups
**South Arvensis**		**2** ***x***		**widespread**	**leaves mostly divided**
*K. ambigua*	*K. arvensis*	2*x*	grasslands and ruderal places	SE Balkan Peninsula	
*K. arvensis*	*K. arvensis*	2*x* [4*x*]	grasslands and ruderal places	most of Europe except for the extreme south	
*K. lucana*	*K. arvensis*	2*x*	forests	S Italy	
*K. macedonica*	*K. arvensis*	2*x*	grasslands and ruderal places	Central Balkan Peninsula	
*K. visianii*	*K. arvensis*	2*x*	grasslands and ruderal places	Central and W Balkan Peninsula	
**Carinthiaca & North Arvensis**		**2** ***x*** **, 4** ***x***		**widespread**	**morphologically heterogeneous with mostly divided leaves**
*K. arvensis*	*K. arvensis*	[2*x*] 4*x*	grasslands and ruderal places	most of Europe except for the extreme south	
*K. carinthiaca*	*K. velutina*	2*x*	natural grasslands and rock cervices, limestone	Austria (E Central Alps)	
*K. dipsacifolia* p.p.	*K. silvatica*	4*x* [6*x*]	forest margins, tall herb communities, meadows	Central Europe [Alps, Central Balkan Peninsula]	
*K. kitaibelii*		4*x*	meadows, wood margins	Central Europe	
*K. norica*	*K. subcanescens*/*persicina*	4*x*	grassy forest understory, rocks	Austria (E Alps)	
*K. pseudolongifolia*		2*x*	alpine rock pastures	W Krkonoše (Czech Republic)	
*K. serpentinicola*		2*x*	serpentine forest clearings and edges	NW Czech Republic	
*K. slovaca*		2*x*	natural grasslands on limestone	central E Slovakia	
**Drymeia & Dinarica**		**2** ***x*** **, 4** ***x***		**widespread**	**mostly undivided leaves; soft indumentum with additional, mostly yellowish setae on basal parts, partly monopodial rhizomes**
*K. csikii*	*K. dinarica*	2*x*	(sub) alpine meadows, tall herb communities	Central and W Balkan Peninsula	
*K. dinarica*	*K. dinarica*	2*x*, 4*x*	(sub) alpine meadows, tall herb communities	Central and W Balkan Peninsula; S Italy	
*K. dipsacifolia* p.p.	*K. silvatica*	4*x* [6*x*]	rocky forest clearings	Central Balkan Peninsula [Alps, Central Europe]	
*K. drymeia*	*K. drymeia*	2*x*, 4*x*	forest and forest margins	Central and SE Europe, N Italy	
*K. sarajevensis*	*K. sarajevensis*	4*x*	forest margins	Central Balkan Peninsula	
*K*. sp. 2		4*x*	serpentine clearings	Central Balkan Peninsula	
**Alpine Dipsacifolia**		**6** ***x***		**regional**	**Undivided, dentate leaves**
*K. dipsacifolia* p.p.	*K. silvatica*	[4*x*] 6*x*	forests, forest margins, tall herb communities	Alps [Central Europe, Central Balkan Peninsula]	
**Longifolia**		**2** ***x*** **, 4** ***x*** **, 6** ***x***		**regional, disjunct**	**often undivided leaves, lanceolate, with entire margins, divided leaves with long terminal segment**
*K. baldensis*	*K. subcanescens*/*persicina*	4*x*	subalpine meadows	N Italy	
*K. fleischmannii*	*K. fleischmannii*	4*x*	thermophilous shrubland	Slovenia	
*K. illyrica* p.p.	*K. arvensis*	2*x*, 4*x* [6*x*]	meadows, pastures, open forests	SE Alps to NW Balkan Peninsula	
*K. longifolia*	*K. longifolia*	2*x*	upper montane to alpine meadows and tall herb communities	S and E Alps, S and E Carpathians	
*K. pancicii*	*K. longifolia*	2*x*	damp mountain meadows	Zlatibor planina (Serbia)	
*K. persicina*	*K. subcanescens*/*persicina*	4*x*	stabilised limestone screes	N Italy	
*K. purpurea* p.p.	*K. arvensis*	2*x*, 4*x*	meadows	SW and S Europe	
*K. ressmannii*	*K. silvatica*	6*x*	forest margins	N Italy	
*K. velutina*	*K. velutina*	2*x*	screes, pastures, forest edges	S Alps	
**Midzorensis**		**2** ***x*** **, 4** ***x***		**regional, continuous**	**undivided lanceolate leaves with entire margins; peduncles glandular or eglandular**
*K. magnifica*		4*x*	subalpine grasslands, pastures	E central Balkan Peninsula	
*K. midzorensis*	*K. longifolia*	2*x*, 4*x*	tall herb communities, rocky pastures	E central Balkan Peninsula	
**Montana**		**2** ***x***		**widespread**	**yellow-flowering; often tall herbs with ovate, undivided to lyrate leaves**
*K. involucrata*	*K. montana*	2*x*	subalpine meadows	Anatolia to Caucasus	
*K. montana*	*K. montana*	2*x*	tall herb communities	Caucasus to Urals	
**SW European**		**2** ***x*** **, 4** ***x*** **, 6** ***x***		**widespread, but disjunct**	**heterogeneous; often undivided leaves, sometimes deeply divided; leaves glabrous to densely hairy**
*K. arvernensis*	*K. silvatica*	4*x*	forest margins, wet meadows	Massif Central (France)	
*K. basaltica*	*K. longifolia*	2*x*	(sub) alpine grasslands on volcanic bedrock	Massif Central (France)	
*K. foreziensis*		4*x*	dwarf shrub-communities		
*K. godetii*	*K. longifolia*	2*x*	wet meadows, fens	Massif Central (France) to Jura Mountains (Switzerland)	
*K. lebrunii*	*K. longifolia*	2*x*	tall herb communities, open forests	Eastern Pyrenees	
*K. legionensis*		4*x*	meadows	N Iberian Peninsula	
*K. nevadensis*	*K. silvatica*	4*x*	tall herb communities, road margins, silicate	Iberian Peninsula	
*K. rupicola*		4*x*, 6*x*	montane meadows, limestone screes	E Iberian Peninsula	
*K. subcanescens*	*K. subcanescens*/*persicina*	2*x*, 4*x*	tall herb communities, (sub) alpine meadows	Western Alps	
*K. subscaposa*		2*x*	grassy and rocky slopes	Iberian Peninsula	
**Xerophytic**		**2** ***x*** **, 4** ***x*** **, 6** ***x***		**widespread**	**usually strongly divided leaves with one to three pairs of lobes, often densely lanuginose to tomentose**
*K. adriatica*		4*x*	rocky limestone slopes, calcareous hillsides, scrub margins	Dalmatia (Croatia)	
*K. albanica*	*K. velutina*	2*x*	dry grasslands	Central W Balkan Peninsula	
*K. arvensis*	*K. arvensis*	2*x*, 4*x*	grasslands and ruderal places	most of Europe except for the extreme south	
*K. calycina*	*K. velutina*	2*x*	mountain grasslands	Central and S Italy, Sicily	
*K. clementii*	*K. dalmatica*	4*x*, 6*x*	dry grasslands	Dalmatia (Croatia)	
*K. collina*	*K. arvensis*	2*x*	dry grasslands and subruderal places	S France and NW Italy?	
*K. dalmatica*	*K. dalmatica*	4*x*	rocky limestone slopes, calcareous hillsides, scrub margines	Dalmatia (Croatia)	
*K. illyrica* p.p.	*K. arvensis*	2*x*, 4*x*, 6*x*	dry grasslands	SE Alps to NW Balkan Peninsula	
*K. mollis*	*K. velutina*	2*x*	mountain grasslands	SW Alps	
*K. pectinata*	*K. dalmatica*	2*x*, 4*x*	dry mountain grasslands	NW Balkan Peninsula (Velebit)	
*K. purpurea* p.p.	*K. arvensis*	2*x*, 4*x*	dry grasslands and ruderal places	SW and S Europe	
*K*. sp. 1		6*x*	upper montane meadows	NW Balkan Peninsula (S Velebit)	
*K. travnicensis*	*K. fleischmannii*	2*x*, 4*x*, 6*x*	dry mountain grasslands	Central NW Balkan Peninsula	
*K. velebitica*	K. *velutina*	2*x*	dry mountain grasslands	NW Balkan Peninsula (Velebit)	

Whether polyploids originated via autopolyploidy or allopolyploidy is unclear due to the weak genetic separation among species. In a separate analysis based on a comprehensive population sampling in *K. drymeia*, both auto- (i.e. within the same genetic lineage) as well as allopolyploid (i.e. between genetic lineages) origins of tetraploids were inferred within the same species [95], suggesting that this might also be the case in other polyploid species of *Knautia*. Recurrent evolution of polyploids is also evident for *K. dipsacifolia*, which falls into three different AFLP groups (Fig. 6). This is consistent with numerous molecular phylogenetic studies, which have demonstrated the recurrent formation of polyploids in many plant groups (e.g. [96–98]). In a geographical context, AFLP differentiation among species as expressed by bootstrap support tends to be less pronounced in central Europe than further south (Additional file 8: Figures S7–S9). This also results in a bad fit of AFLP-based relationships and current taxonomy as accessions of widespread species such as *K. arvensis* or *K. dipsacifolia* fail to cluster. Such pattern likely reflects a more dynamic glacial and postglacial history in Central Europe as compared to a more static scenario in Southern Europe, which also emerged previously in intraspecific phylogeographic studies (e.g., [99–101]). Although populations with mixed ploidy levels are known in *Knautia* [102], they were not found in our previous study [25] including 381 populations. This suggests that intrapopulational cytotype mixture is rare and likely restricted to primary contact zones, i.e. areas of recent polyploidisation events [103], as shown for the locally endemic *K. serpentinicola* [40, 104]. Nevertheless, the large geographical distribution of many polyploids indicates their successful and stable long-term establishment.

## Conclusions

Altogether, the heteroploid, species-rich *K*. sect. *Trichera* is a prime example of rapid diversification mostly taking place during the Pliocene and Pleistocene. Addition of polyploids to a previously established phylogenetic framework for diploids [24] revealed that polyploids have originated mostly within the groups of diploid species rather than between groups. Generally, discrepancies remain between the circumscription of the species groups proposed here on the basis of AFLP data and the formerly recognised groups defined by morphological, karyological and eco-geographical criteria [21]. In addition, several species appear in two or three of the weakly defined genetic groups. As most *Knautia* species—exceptions being a few forest understory herbs—are light demanding inhabitants of various types of grassland or forb communities, it appears likely that forest advance during warm stages of the Pleistocene has led to the separation of gene pools [91]. Such separation was terminated by the expansion of grasslands during cold or dry periods making secondary contacts possible. Numerous cycles of habitat fragmentation and subsequent reconnections likely promoted interspecific hybridisation (as detected between *K. arvensis* and *K. carinthiaca* by Čertner et al. [91]) and eventually polyploidisation and resulted in the highly complex and heterogeneous genetic constitution of several *Knautia* species. Extensive haplotype sharing and unresolved phylogenetic relationships suggest that these processes occurred rapidly and extensively within sect. *Trichera*. Although our taxonomically almost complete phylogeny revealed general patterns of the genus’ evolution, the weak and partly contradicting phylogenetic structure renders it premature to take taxonomic decisions. On the contrary, it appears likely that (*i*) the dynamic polyploid evolution of sect. *Trichera*, (*ii*) the lack of crossing barriers within ploidy levels likely supported by the conserved floral morphology, (*iii*) the highly variable leaf morphology and (*iv*) the unstable indumentum composition prevent establishing a well-founded taxonomic framework. All this is in perfect agreement with the section’s reputation as one of the most intricate taxa of the European flora.

## Additional files

Additional file 1: Table S1. Voucher information and GenBank accession numbers. More information can be retrieved from http://www.uibk.ac.at/botany/balkbiodiv/?BALKBIODIV under “Sampling sites” (XLS 166 kb)
Additional file 2: Figure S1. Bayesian consensus phylogram of Dipsacoideae focussing on *Knautia* based on plastid *pet*N(*ycf*6)-*psb*M sequences. Values above and below branches are parsimony bootstrap values > 50 and posterior probabilities derived from Bayesian analysis > 0.80, respectively. Population IDs, provenance countries and ploidy are printed after the species names. The print colour follows the haplotype groups in Fig. 2a. Terminal branches are coloured according to ploidy level: black, diploid; red, tetraploid; green, hexaploid (PDF 1388 kb)
Additional file 3: Figure S2. Bayesian consensus phylogram of Dipsacoideae focussing on *Knautia* based on Internal Transcribed Spacer (ITS) sequences. Values above and below branches are parsimony bootstrap values > 50 and posterior probabilities derived from Bayesian analysis > 0.80, respectively. The colour of terminal branches represents ploidy: black, diploid; red, tetraploid; green, hexaploid (PDF 1386 kb)
Additional file 4: Figure S3. Internal Transcribed Spacer (ITS) variation in populations of species of *Knautia* sect. *Trichera* showing correlation with the AFLP groups presented in Fig. 6 and the groups of plastid DNA haplotypes shown in Fig. 2. A, relationships are visualised as NeighbourNet diagrams based on uncorrected P distances. Colouring of population IDs corresponds to the groups of plastid DNA haplotypes shown in Fig. 2. The colour shading follows major AFLP groups corresponding to Figures S7–S9 within the Additional file 8. Deviations result from the necessity of uniting the previously separated Drymeia and Dinarica Groups as well as the Carinthiaca and North Arvensis Groups. B, NeighbourNet diagram of the diploid taxa as identified previously by Rešetnik et al. [24] (PDF 1432 kb)
Additional file 5: Figure S4. Internal Transcribed Spacer (ITS) variation in some species of *Knautia* sect. *Trichera*, illustrating that many species host unrelated ribotypes. For each taxon, position of its ribotypes in the NeighbourNet (fully presented in Additional file 4: Figure S3) is indicated by coloured dots (PDF 1587 kb)
Additional file 6: Figure S5. Relationships among populations of 51 species of *Knautia* sect. *Trichera* visualised by a Neighbour-joining tree based on Nei-Li distances derived from AFLP data. The tree is rooted with *K. integrifolia* from sect. *Tricheroides*. Bootstrap support values (based on 1000 replicates) above 50 % are given for branches with ≥ 3 terminals. Symbols for the species correspond to those used in Fig. 1. Separate labelling at the tips of branches was used when individuals of different taxa were intermixed. Population numbers given to the right of the species’ names correspond to Supplementary Table S1. Species groups given at the right margin are detailed in Table 3 and the Supplementary File S7 (PDF 901 kb)
Additional file 7: Figure S6. Amplified Fragment Length Polymorphism (AFLP) variation in 251 populations of 51 species of *Knautia* sect. *Trichera* visualised as NeighbourNet diagram based on uncorrected P distances. The colours of individual branches indicate ten genetic clusters as identified by K-means clustering. This is an enlargeable version of Fig. 6 with labelling of terminal splits with species names and population IDs (PDF 1493 kb)
Additional file 8: Informal classification of diploid and polyploid *Knautia* (containing Figures S7–S9) (PDF 664 kb)

## References

[1] Soltis DE, Albert VA, Leebens-Mack J, Bell CD, Paterson AH (2009). Polyploidy and angiosperm diversification. Am J Bot.

[2] Wood TE, Takebayashi N, Barker MS, Mayrose I, Greenspoon PB, Rieseberg LH (2009). The frequency of polyploid speciation in vascular plants. Proc Natl Acad Sci U S A.

[3] Husband BC, Baldwin SJ, Suda J, Leitch IJ, Greilhuber J, Doležel J, Wendel JF (2013). The incidence of polyploidy in natural plant populations: major patterns and evolutionary processes. Plant Genome Diversity.

[4] Madlung A (2013). Polyploidy and its effect on evolutionary success: old questions revisited with new tools. Heredity.

[5] Otto SP, Whitton J (2000). Polyploid incidence and evolution. Annu Rev Genet.

[6] Popp M, Erixon P, Eggens F, Oxelman B (2005). Origin and evolution of a circumpolar polyploid species complex in *Silene* (Caryophyllaceae) inferred from low copy nuclear RNA Polymerase introns, rDNA, and chloroplast DNA. Syst Bot.

[7] Marhold K, Lihova J (2006). Polyploidy, hybridization and reticulate evolution: lessons from the Brassicaceae. Pl Syst Evol.

[8] Brysting AK, Mathiesen C, Marcussen T (2011). Challenges in polyploid phylogenetic reconstruction: A case story from the arctic-alpine *Cerastium alpinum* complex. Taxon.

[9] Stebbins GL (1971). Chromosomal evolution in higher plants.

[10] Chapman MA, Abbott RJ (2010). Introgression of fitness genes across a ploidy barrier. New Phytol.

[11] Leitch IJ, Bennett MD (2004). Genome downsizing in polyploid plants. Biol J Linn Soc.

[12] Adams KL, Wendel JF (2005). Polyploidy and genome evolution in plants. Curr Opin Plant Biol.

[13] Johnston JS, Pepper AE, Hall AE, Chen ZJ, Hodnett G, Drabek J (2005). Evolution of genome size in Brassicaceae. Ann Bot.

[14] Parisod C, Holderegger R, Brochmann C (2010). Evolutionary consequences of autopolyploidy. New Phytol.

[15] Sonnleitner M, Flatscher R, Escobar García P, Rauchová J, Suda J (2010). Distribution and habitat segregation on different spatial scales among diploid, tetraploid and hexaploid cytotypes of *Senecio carniolicus* (Asteraceae) in the Eastern Alps. Ann Bot.

[16] Balao F, Herrera J, Talavera S (2011). Phenotypic consequences of polyploidy and genome size at the microevolutionary scale: a multivariate morphological approach. New Phytol.

[17] Weiss-Schneeweiss H, Emadzade K, Jang T-S, Schneeweiss GM (2013). Evolutionary consequences, constraints and potential of polyploidy in plants. Cytogenet Genome Res.

[18] Mayrose I, Zhan SH, Rothfels CJ, Magnuson-Ford K, Barker MS, Rieseberg LH (2011). Recently formed polyploid plants diversify at lower rates. Science.

[19] Arrigo N, Barker MS (2012). Rarely successful polyploids and their legacy in plant genomes. Curr Opin Plant Biol.

[20] MacIntyre GT (1967). Foramen pseudovale and quasi-mammals. Evolution.

[21] Ehrendorfer F (1962). Beiträge zur Phylogenie der Gattung *Knautia* (Dipsacaceae), I. Cytologische Grundlagen und allgemeine Hinweise. Österr Botsch Zagreb.

[22] Ehrendorfer F (1962). Cytotaxonomische Beiträge zur Genese der mitteleuropäischen Flora und Vegetation. Ber Deut Bot Ges.

[23] Carlson SE, Mayer V, Donoghue MJ (2009). Phylogenetic relationships, taxonomy, and morphological evolution in Dipsacaceae (Dipsacales) inferred by DNA sequence data. Taxon.

[24] Rešetnik I, Frajman B, Bogdanović S, Ehrendorfer F, Schönswetter P (2014). Disentangling relationships among the diploid members of the intricate genus *Knautia* (Caprifoliaceae, Dipsacoideae). Mol Phylogenet Evol.

[25] Frajman B, Rešetnik I, Weiss-Schneeweiss H, Ehrendorfer F, Schönswetter P (2015). Cytotype diversity and genome size variation in *Knautia* (Caprifoliaceae, Dipsacoideae). BMC Evol Biol.

[26] Drummond CS, Eastwood RJ, Miotto ST, Hughes CE (2012). Multiple continental radiations and correlates of diversification in *Lupinus* (Leguminosae): testing for key innovation with incomplete taxon sampling. Syst Biol.

[27] Jabbour F, Renner SS (2012). A phylogeny of Delphinieae (Ranunculaceae) shows that *Aconitum* is nested within *Delphinium* and that Late Miocene transitions to long life cycles in the Himalayas and Southwest China coincide with bursts in diversification. Mol Phylogent Evol.

[28] Bell CD, Mavrodiev EV, Soltis PS, Calaminus AK, Albach DC, Cellinese N (2012). Rapid diversification of *Tragopogon* and ecological associates in Eurasia. J Evol Biol.

[29] Valente LM, Savolainen V, Vargas P (2010). Unparalleled rates of species diversification in Europe. Proc R Soc B.

[30] Ehrendorfer F (1981). Neue Beiträge zur Karyosystematik und Evolution der Gattung *Knautia* (Dipsacaceae) in den Balkanländern. Bot Jahrb Syst.

[31] Bussell JD, Waycott M, Chappill JA (2005). Arbitrarily amplified DNA markers as characters for phylogenetic inference. Persp Plant Ecol Evol Syst.

[32] Althoff DM, Gitzendanner MA, Segraves KA (2007). The utility of amplified fragment length polymorphisms in phylogenetics: a comparison of homology within and between genomes. Syst Biol.

[33] Tremetsberger K, Stuessy TF, Kadlec G, Urtubey E, Baeza CM, Beck SG, Valdebenito HA, Ruas CDF, Matzenbacher NI. AFLP phylogeny of South American species of *Hypochaeris* (Asteraceae, Lactuceae) Syst Bot. 2006;31:610–26.

[34] Koopman WJM, Wissemann V, De Cock K, Van Huylenbroeck J, De Riek J, Sabatino GJH (2008). AFLP markers as a tool to reconstruct complex relationships: a case study in *Rosa* (Rosaceae). Am J Bot.

[35] Bardy KE, Albach DC, Schneeweiss GM, Fischer MA, Schönswetter P (2010). Disentangling phylogeography, polyploid evolution and taxonomy of a woodland herb (*Veronica chamaedrys* group, Plantaginaceae s.l.) in southeastern Europe. Mol Phylogenet Evol.

[36] Greiner R, Vogt R, Oberprieler C (2013). Evolution of the polyploid north-west Iberian *Leucanthemum pluriflorum* clan (Compositae, Anthemideae) based on plastid DNA sequence variation and AFLP fingerprinting. Ann Bot.

[37] Ehrendorfer F. *Knautia* L. In: Tutin T, Heywood VH, Burges NA, Moore DM, Valentine DH, Walters SM, Webb DA, editors. Flora Europaea. vol 4. Cambridge: Cambridge University Press; 1976. p. 60–7.

[38] Diklić N. Dipsacaceae B. Juss. In: Josifović M, editor. Flora SR Srbije. Vol. 5. Beograd: Srpska Akademija Nauka i Umetnosti; 1973. p. 536–84.

[39] Štěpánek J (1983). Eine neue Art der Gattung *Knautia* (Dipsacaceae) aus Westkarpaten. Preslia.

[40] Kolář F, Kaplan Z, Suda J, Štech M (2015). Populations of *Knautia* in ecologically distinct refugia on the Hercynian massif belong to two endemic species. Preslia.

[41] Devesa JA. *Knautia* L. In: Devesa JA, Gonzalo R, Herrero A, editors. Flora Iberica. Plantas vasculares de la Península Ibérica e Islas Baleares, vol. XV, Rubiaceae–Dipsacaceae. Madrid: Real Jardín Botánico, CSIC; 2007. p. 286–305.

[42] Matthews VA. *Knautia* L. In: Davis PH, editor. Flora of Turkey and East Aegean Islands, 4. Edinburgh:, University Press; 1972. p. 598–601.

[43] Shaw J, Lickey EB, Beck JT, Farmer SB, Liu W, Miller J (2005). The tortoise and the hare II: Relative utility of 21 noncoding chloroplast DNA sequences for phylogenetic analysis. Am J Bot.

[44] Sun Y, Skinner DZ, Liang GH, Hulbert SH (1994). Phylogenetic analysis of *Sorghum* and related taxa using internal transcribed spacers of nuclear ribosomal DNA. Theor Appl Genet.

[45] Arrigo N, Tuszynski JW, Ehrich D, Gerdes T, Alvarez N. Evaluating the impact of scoring parameters on the structure of intra-specific genetic variation using RawGeno, an R package for automating AFLP scoring. BMC Bioinformatics. 2009; doi:10.1186/1471-2105-10-33. 10.1186/1471-2105-10-33 PMC2656475 19171029

[46] R Development Core Team. R: A language and environment for statistical computing. R Foundation for Statistical Computing, Vienna, Austria. 2012. http://www.R-project.org. Accessed 08 Sept 2015.

[47] Bonin A, Bellemain E, Bronken Eidesen P, Pompanon F, Brochmann C, Taberlet P (2004). How to track and assess genotyping errors in population genetics studies. Mol Ecol.

[48] Drummond AJ, Ashton B, Buxton S, Cheung M, Cooper A, Duran C, Field M, Heled J, Kearse M, Markowitz S, Moir R, Stones-Havas S, Sturrock S, Thierer T, Wilson A. Geneious v5.4. 2011. Available from http://www.geneious.com/10.1093/bioinformatics/bts199 PMC3371832 22543367

[49] Swofford DL. PAUP. Phylogenetic Analysis Using Parsimony (*and Other Methods), ver. 4.0 Beta 10. Sunderland: Sinauer Associates; 2002.

[50] Ronquist F, Teslenko M, van der Mark P, Ayres DL, Darling A, Höhna S (2012). MrBayes 3.2: Efficient Bayesian phylogenetic inference and model choice across a large model space. Syst Biol.

[51] Nylander JAA. MrAIC.pl. Program distributed by the author. Uppsala: Evolutionary Biology Centre, Uppsala University; 2004.

[52] Bryant D, Moulton V (2004). Neighbour-net: an agglomerative method for the construction of phylogenetic networks. Mol Biol Evol.

[53] Huson DH (1998). SplitsTree: a program for analyzing and visualizing evolutionary data. Bioinformatics.

[54] Huson DH, Bryant D (2006). Application of phylogenetic networks in evolutionary studies. Mol Biol Evol.

[55] Clement M, Posada D, Crandall KA (2000). TCS: a computer program to estimate gene genealogies. Mol Ecol.

[56] Drummond AJ, Rambaut A (2007). BEAST: Bayesian evolutionary analysis by sampling trees. BMC Evol Biol.

[57] Carlson SE, Linder HP, Donoghue MJ (2012). The historical biogeography of *Scabiosa* (Dipsacaceae): implications for Old World plant disjunctions. J Biogeogr.

[58] Drummond AJ, Ho SYW, Phillips MJ, Rambaut A (2006). Relaxed phylogenetics and dating with confidence. PLoS Biol.

[59] Rambaut A, Suchard MA, Xie D, Drummond AJ. Tracer v1.6. 2014. http://beast.bio.ed.ac.uk/tracer.

[60] Rambaut A. FigTree 1.4.2. Computer program and documentation distributed by the author, 2014. http://tree.bio.ed.ac.uk/

[61] Lemey P, Rambaut A, Welch JJ, Suchard MA (2010). Phylogeography takes a relaxed random walk in continuous space and time. Mol Biol Evol.

[62] Drummond AJ, Suchard MA, Xie D, Rambaut A (2012). Bayesian phylogenetics with BEAUti and the BEAST 1.7. Mol Biol Evol.

[63] Drummond AJ, Rambaut A, Shapiro B, Pybus OG (2005). Bayesian coalescent inference of past population dynamics from molecular sequences. Mol Biol Evol.

[64] Bielejec F, Rambaut A, Suchard MA, Lemey P. SPREAD: spatial phylogenetic reconstruction of evolutionary dynamics. Computer program and documentation distributed by the author, 2011. website: URL http://www.kuleuven.be/aidslab/phylogeography/SPREAD.html. 10.1093/bioinformatics/btr481 PMC3187652 21911333

[65] Magallón S, Sanderson MJ (2001). Absolute diversification rates in Angiosperm clades. Evolution.

[66] Harmon L, Weir JT, Brock CD, Glor RE, Challenger W (2008). GEIGER: investigating evolutionary radiations. Bioinformatics.

[67] Nei M, Li WH (1979). Mathematical model for studying genetic variation in terms of restriction endonucleases. Proc Natl Acad Sci U S A.

[68] Van de Peer Y, De Wachter R (1997). Construction of evolutionary distance trees with TREECON for Windows: accounting for variation in nucleotide substitution rate among sites. Comput Appl Biosci.

[69] Hartigan JA, Wong MA (1979). A K-means clustering algorithm. Appl Stat.

[70] Arrigo N, Felber F, Parisod C, Buerki S, Alvarez N (2010). Origin and expansion of the allotetraploid *Aegilops geniculata*, a wild relative of wheat. New Phytol.

[71] Burnier J, Buerki S, Arrigo N, Kuepfer P, Alvarez N (2009). Genetic structure and evolution of Alpine polyploid complexes: *Ranunculus kuepferi* (Ranunculaceae) as a case study. Mol Ecol.

[72] Kolář F, Fér T, Štech M, Trávníček T, Dušková E, Schönswetter P (2012). Bringing together evolution on serpentine and polyploidy: spatiotemporal history of the ecologically differentiated diploid-tetraploid complex of *Knautia arvensis* (Dipsacaceae). PLoS One.

[73] Mayer V, Ehrendorfer F (2013). The phylogenetic position of *Pterocephalidium* and the new African genus *Pterothamnus* within an improved classification of Dipsacaceae. Taxon.

[74] Myers N, Mittermeier RA, Mittermeier CG, da Fonseca GAB, Kent J (2000). Biodiversity hotspots for conservation priorities. Nature.

[75] Greuter W. Botanical diversity, endemism, rarity, and extinction in the Mediterranean area: an analysis based on the published volumes of Med-Checklist. Bot Chron (Patras). 1991;10:63–79.

[76] Nieto Feliner G (2014). Patterns and processes in plant phylogeography in the Mediterranean Basin. A review. Perspect Plant Ecol Evol Syst.

[77] Mansion G, Selvi F, Guggisberg A, Conti E (2009). Origin of Mediterranean insular endemics in the Boraginales: integrative evidence from molecular dating andancestral area reconstruction. J Biogeogr.

[78] Roquet C, Sanmartín I, Garcia-Jacas N, Sáez L, Susanna A, Wikström N (2009). Reconstructing the history of Campanulaceae with a Bayesian approach to molecular dating and dispersal-vicariance analyses. Mol Phylogenet Evol.

[79] Barres L, Sanmartín I, Anderson CL, Susanna A, Buerki S, Galbany-Casals M (2013). Reconstructing the evolution and biogeographic history of tribe *Cardueae* (Compositae). Am J Bot.

[80] Hand R, Hadjikyriakou GN, Christodoulou CS, Frajman B (2015). Multiple origins of dendroid shrubs in the Eastern Mediterranean *Euphorbia hierosolymitana* group (Euphorbiaceae) with description of a new species, *Euphorbia lemesiana*, from Cyprus. Bot J Linn Soc.

[81] Rögl F (1999). Mediterranean and Paratethys. Facts and hypotheses of an Oligocene to Miocene paleogeography (short overview). Geol Carpath.

[82] Ehrendorfer F. *Knautia*. In: Pignatti S, editor. Flora d’Italia, vol. 2. Bologna: Edagricole; 1982. p. 664–70.

[83] Hsü KJ, Montadert L, Bernoulli D, Cita MB, Erickson A, Garrison RE (1977). History of the Mediterranean salinity crisis. Nature.

[84] Krijgsman W, Hilgen FJ, Raffi I, Sierro FJ, Wilson DS (1999). Chronology, causes and progression of the Messinian Salinity Crisis. Nature.

[85] Meulenkamp JE, Sissingh W (2003). Tertiary palaeogeography and tectonostratigraphic evolution of the Northern and Southern Peri-Tethys platforms and the intermediate domains of the African–Eurasian convergent plate boundary zone. Palaeogeogr Palaeoclimatol Palaeoecol.

[86] Champagnac J-D, Schlunegger F, Norton K, von Blanckenburg F, Abbühl LM, Schwabb M (2009). Erosion-driven uplift of the modern Central Alps. Tectonophysics.

[87] Combourieu-Nebout N (1993). Vegetation response to upper Pliocene glacial/interglacial cyclicity in the central Mediterranean. Quat Res.

[88] Kadereit JW, Griebeler EM, Comes HP (2004). Quaternary diversification in European alpine plants: pattern and process. Philos Trans R Soc Lond B.

[89] Schönswetter P, Stehlik I, Holderegger R, Tribsch A (2005). Molecular evidence for glacial refugia of mountain plants in the European Alps. Mol Ecol.

[90] Winkler M, Tribsch A, Paun O, Englisch T, Schönswetter P (2010). Pleistocene distribution shifts were accompanied by breeding system divergence within *Hornungia alpina* (Brassicaceae) in the Alps. Mol Phylogenet Evol.

[91] Čertner M, Kolář F, Schönswetter P, Frajman B (2015). Does hybridization with a widespread congener threaten the long-term persistence of the Eastern Alpine rare local endemic *Knautia carinthiaca*?. Ecol Evol.

[92] Griffiths HI, Kryštufek B, Reed JM. Balkan Biodiversity – Pattern and process in the European hotspot. Dordrecht: Kluwer Academic Publishers; 2004.

[93] Hewitt GM. Mediterranean peninsulas: the evolution of hotspots. In: Zachos FE, Habel JC, editors. Biodiversity hotspots. Berlin: Springer; 2011. p. 123–47.

[94] Kropf M, Kadereit JW, Comes HP (2002). Late Quaternary distributional stasis in the submediterranean mountain plant *Anthyllis montana* L. (Fabaceae) inferred from ITS sequences and amplified fragment length polymorphism markers. Mol Ecol.

[95] Rešetnik I, Frajman B, Schönswetter P (2016). Heteroploid *Knautia drymeia* includes *K. gussonei* and cannot be separated into diagnosable subspecies. Am J Bot.

[96] Brochmann C, Soltis PS, Soltis DE (1992). Recurrent formation and polyphyly of Nordic polyploids in *Draba* (Brassicaceae). Am J Bot.

[97] Soltis DE, Soltis PS (1999). Polyploidy: recurrent formation and genome evolution. Trends Ecol Evol.

[98] Soltis DE, Buggs RJA, Barbazuk WB, Chamala S, Chester M, Gallagher JP, Soltis PS, Soltis DE (2012). The early stages of polyploidy: rapid and repeated evolution in *Tragopogon*. Polyploidy and genome evolution.

[99] Hampe A, Petit RJ (2005). Conserving biodiversity under climate change: the rear edge matters. Ecol Lett.

[100] Ehrich D, Gaudeul M, Assefa A, Koch MA, Mummenhof K, Nemomissa S (2007). Intrabiodiv-Consortium, Brochmann C. Genetic consequences of Pleistocene range shifts: Contrast between the Arctic, the Alps and the East African mountains. Mol Ecol.

[101] Ronikier M, Schneeweiss GM, Schönswetter P (2012). The extreme disjunction between Beringia and Europe in the arctic-alpine *Ranunculus glacialis* s. l. does not coincide with the deepest genetic split – a story of the importance of temperate mountain ranges in arctic-alpine phylogeography. Mol Ecol.

[102] Kolář F, Štech M, Trávníček P, Rauchová J, Urfus T, Vít P (2009). Towards resolving the *Knautia arvensis* agg. (Dipsacaceae) puzzle: primary and secondary contact zones and ploidy segregation at landscape and microgeographic scales. Ann Bot.

[103] Petit C, Bretagnolle F, Felber F (1999). Evolutionary consequences of diploid polyploid hybrid zones in wild species. Trends Ecol Evol.

[104] Hanzl M, Kolář F, Nováková D, Suda J (2014). Nonadaptive processes governing early stages of polyploid evolution: Insights from a primary contact zone of a relict serpentine *Knautia arvensis* (Caprifoliaceae). Am J Bot.

