# Supplementary material for: Patterns of rapid diversification in heteroploid Knautia sect. Trichera (Caprifoliaceae, Dipsacoideae), one of the most intricate taxa of the European flora
Source: BMC Evol Biol. 2016 Oct 10;16:204. doi: 10.1186/s12862-016-0773-2 (PMC5057222; doi:10.1186/s12862-016-0773-2)
Supplement: Additional file 2: Figure S1. — Bayesian consensus phylogram of Dipsacoideae focussing on Knautia based on plastid petN(ycf6)-psbM sequences. Values above and below branches are parsimony bootstrap values > 50 and posterior probabilities derived from Bayesian analysis > 0.80, respectively. Population IDs, provenance countries and ploidy are printed after the species names. The print colour follows the haplotype groups in Fig. 2a. Terminal branches are coloured according to ploidy level: black, diploid; red, tetraploid; green, hexaploid (PDF 1388 kb) [file 12862_2016_773_MOESM2_ESM.pdf]

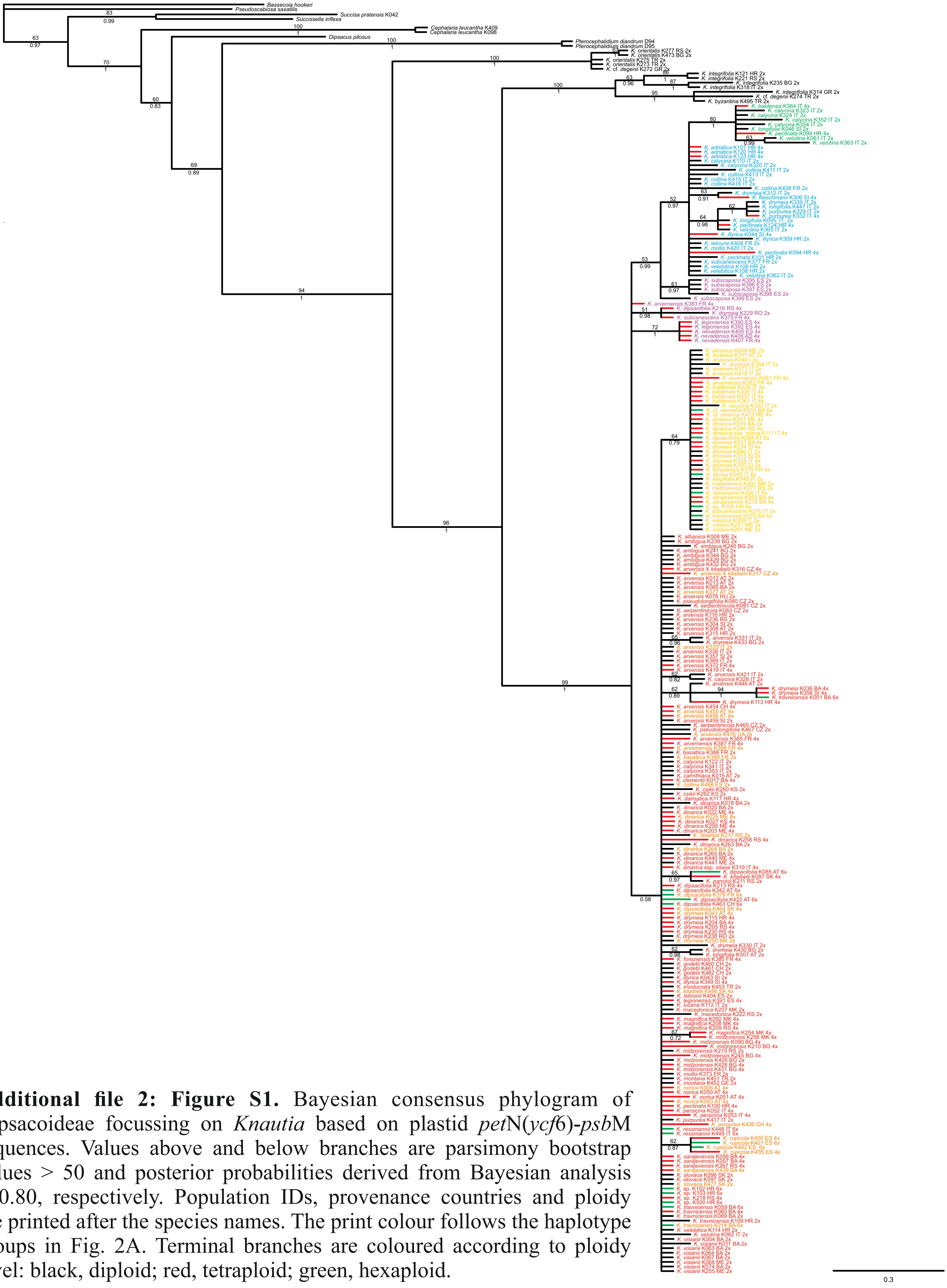

Additional file 2: Figure S1. Bayesian consensus phylogram of Dipsacoideae focussing on *Knautia* based on plastid *pet*N(*ycf*6)-*psb*M sequences. Values above and below branches are parsimony bootstrap values > 50 and posterior probabilities derived from Bayesian analysis > 0.80, respectively. Population IDs, provenance countries and ploidy are printed after the species names. The print colour follows the haplotype groups in Fig. 2A. Terminal branches are coloured according to ploidy level: black, diploid; red, tetraploid; green, hexaploid.
