# Supplementary figures and images for: Patterns of rapid diversification in heteroploid Knautia sect. Trichera (Caprifoliaceae, Dipsacoideae), one of the most intricate taxa of the European flora
Source: BMC Evol Biol. 2016 Oct 10;16:204. doi: 10.1186/s12862-016-0773-2 (PMC5057222; doi:10.1186/s12862-016-0773-2)

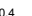

Supplement: Additional file 3: Figure S2. — Bayesian consensus phylogram of Dipsacoideae focussing on Knautia based on Internal Transcribed Spacer (ITS) sequences. Values above and below branches are parsimony bootstrap values > 50 and posterior probabilities derived from Bayesian analysis > 0.80, respectively. The colour of terminal branches represents ploidy: black, diploid; red, tetraploid; green, hexaploid (PDF 1386 kb) [file 12862_2016_773_MOESM3_ESM.pdf]

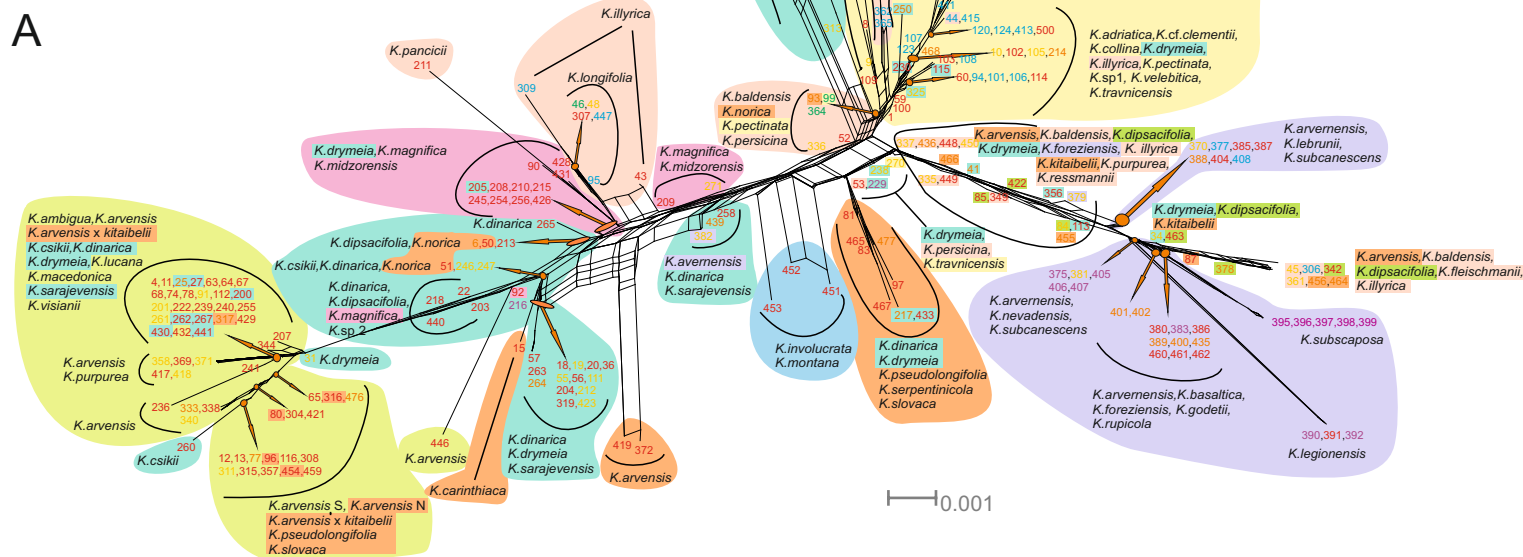

Supplement: Additional file 4: Figure S3. — Internal Transcribed Spacer (ITS) variation in populations of species of Knautia sect. Trichera showing correlation with the AFLP groups presented in Fig. 6 and the groups of plastid DNA haplotypes shown in Fig. 2. A, relationships are visualised as NeighbourNet diagrams based on uncorrected P distances. Colouring of population IDs corresponds to the groups of plastid DNA haplotypes shown in Fig. 2. The colour shading follows major AFLP groups corresponding to Figures S7–S9 within the Additional file 8. Deviations result from the necessity of uniting the previously separated Drymeia and Dinarica Groups as well as the Carinthiaca and North Arvensis Groups. B, NeighbourNet diagram of the diploid taxa as identified previously by Rešetnik et al. [24] (PDF 1432 kb) [file 12862_2016_773_MOESM4_ESM.pdf]
