# Supplementary material for: Patterns of rapid diversification in heteroploid Knautia sect. Trichera (Caprifoliaceae, Dipsacoideae), one of the most intricate taxa of the European flora
Source: BMC Evol Biol. 2016 Oct 10;16:204. doi: 10.1186/s12862-016-0773-2 (PMC5057222; doi:10.1186/s12862-016-0773-2)
Supplement: Additional file 5: Figure S4. — Internal Transcribed Spacer (ITS) variation in some species of Knautia sect. Trichera, illustrating that many species host unrelated ribotypes. For each taxon, position of its ribotypes in the NeighbourNet (fully presented in Additional file 4: Figure S3) is indicated by coloured dots (PDF 1587 kb) [file 12862_2016_773_MOESM5_ESM.pdf]

Additional file 5: Figure S4. Internal Transcribed Spacer (ITS) variation in some species of *Knautia* sect. *Trichera* illustrating that many species host unrelated ribotypes. For each taxon, position of its ribotypes in the NeighbourNet (fully presented in Additional file 4: Figure S3) is indicated by coloured dots.

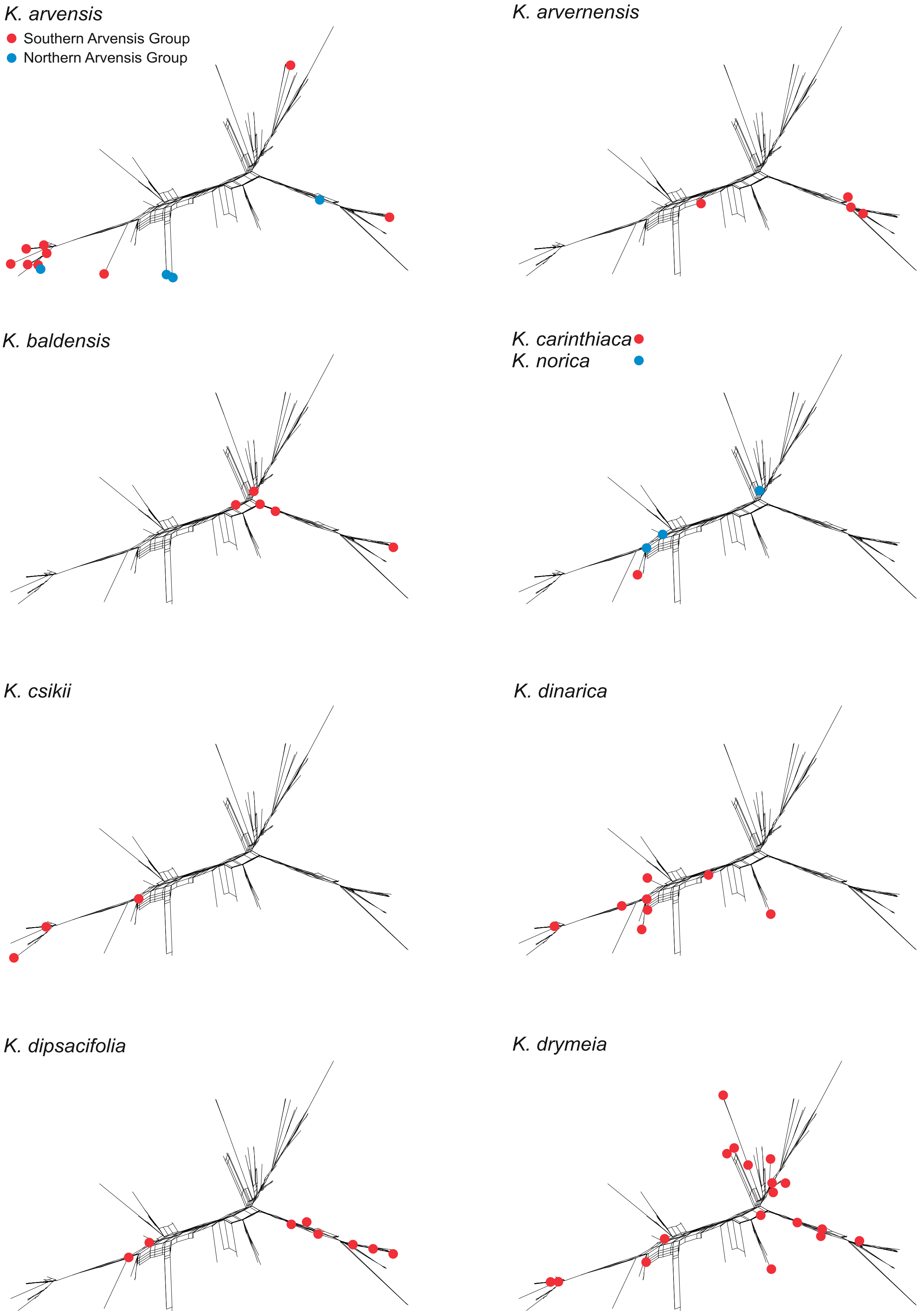

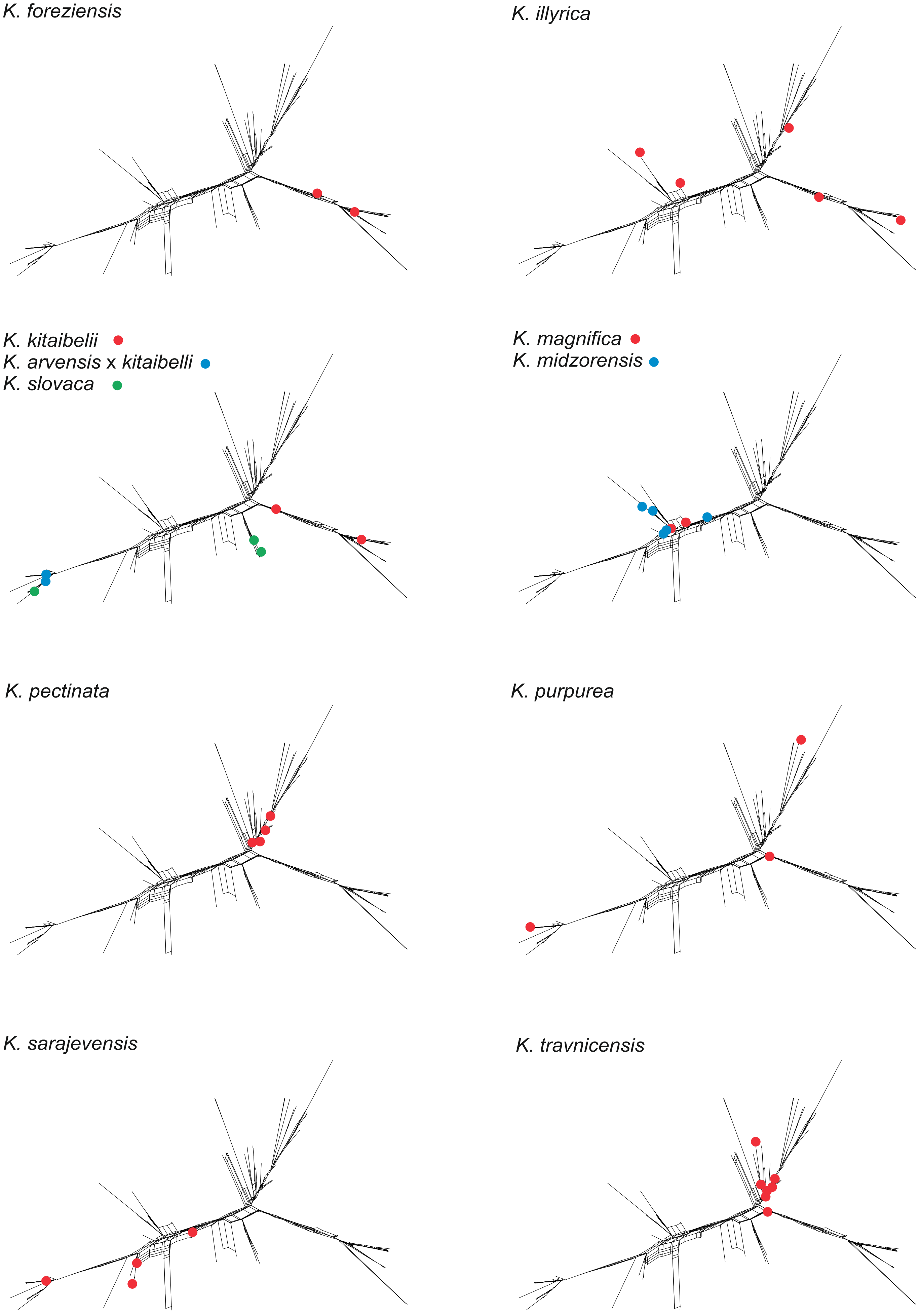
K. foreziensis
K. illyrica
K. kitaibelii
K. arvensis x kitaibelli
K. slovaca
K. magnifica
K. midzorensis
K. pectinata
K. purpurea
K. sarajevensis
K. travnicensis
