# Supplementary material for: Patterns of rapid diversification in heteroploid Knautia sect. Trichera (Caprifoliaceae, Dipsacoideae), one of the most intricate taxa of the European flora
Source: BMC Evol Biol. 2016 Oct 10;16:204. doi: 10.1186/s12862-016-0773-2 (PMC5057222; doi:10.1186/s12862-016-0773-2)
Supplement: Additional file 6: Figure S5. — Relationships among populations of 51 species of Knautia sect. Trichera visualised by a Neighbour-joining tree based on Nei-Li distances derived from AFLP data. The tree is rooted with K. integrifolia from sect. Tricheroides. Bootstrap support values (based on 1000 replicates) above 50 % are given for branches with ≥ 3 terminals. Symbols for the species correspond to those used in Fig. 1. Separate labelling at the tips of branches was used when individuals of different taxa were intermixed. Population numbers given to the right of the species’ names correspond to Supplementary Table S1. Species groups given at the right margin are detailed in Table 3 and the Supplementary File S7 (PDF 901 kb) [file 12862_2016_773_MOESM6_ESM.pdf]

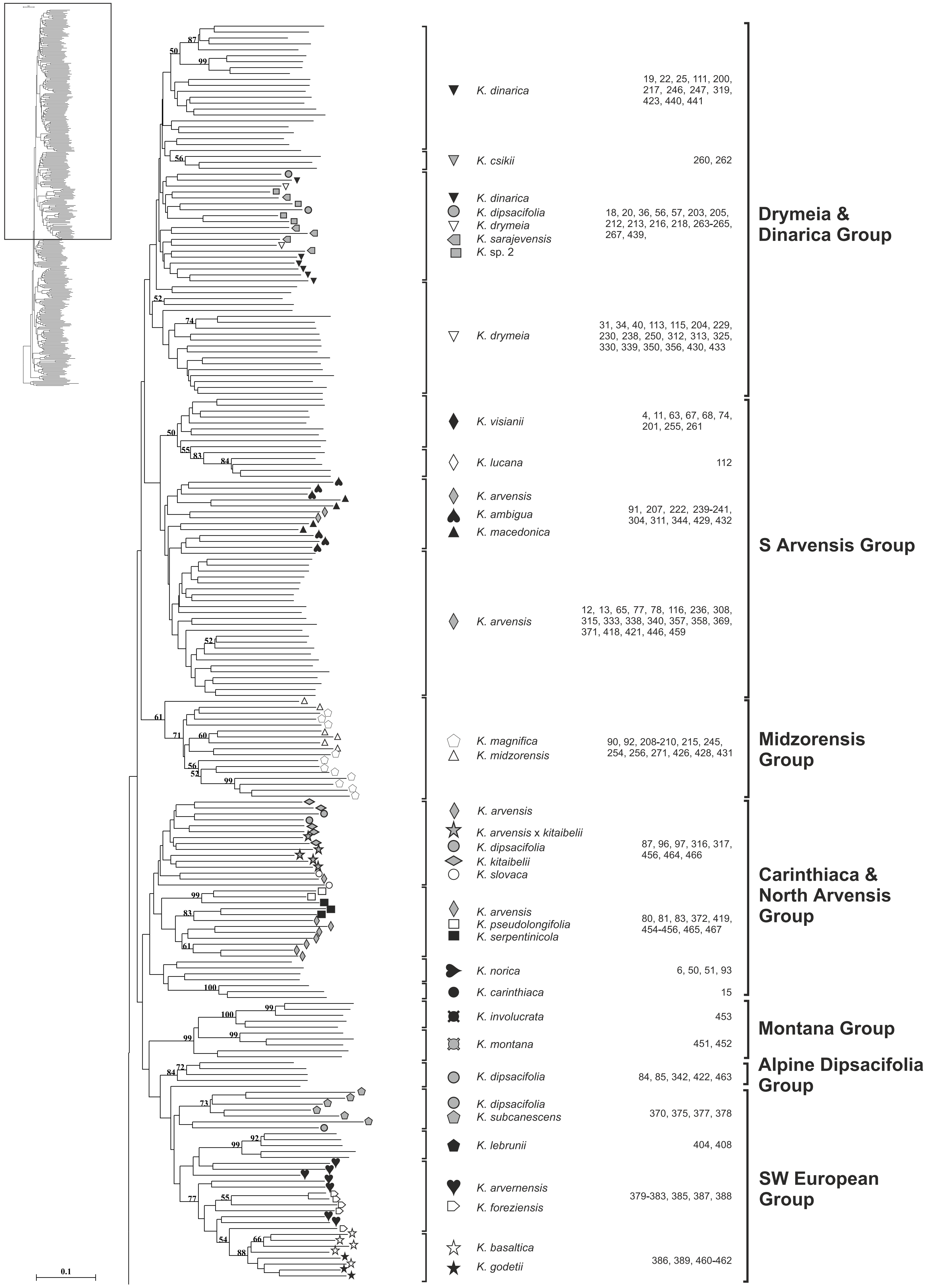
K. dinarica
19, 22, 25, 111, 200, 217, 246, 247, 319, 423, 440, 441
K. csikii
260, 262
K. dinarica
K. dipsacifolia
K. drymeia
K. sarajevensis
K. sp. 2
18, 20, 36, 56, 57, 203, 205, 212, 213, 216, 218, 263-265, 267, 439,
K. drymeia
31, 34, 40, 113, 115, 204, 229, 230, 238, 250, 312, 313, 325, 330, 339, 350, 356, 430, 433
Drymeia & Dinarica Group
K. visianii
4, 11, 63, 67, 68, 74, 201, 255, 261
K. lucana
112
K. arvensis
K. ambigua
K. macedonica
91, 207, 222, 239-241, 304, 311, 344, 429, 432
K. arvensis
12, 13, 65, 77, 78, 116, 236, 308, 315, 333, 338, 340, 357, 358, 369, 371, 418, 421, 446, 459
S Arvensis Group
K. magnifica
K. midzorensis
90, 92, 208-210, 215, 245, 254, 256, 271, 426, 428, 431
Midzorensis Group
K. arvensis
K. arvensis x kitaibelii
K. dipsacifolia
K. kitaibelii
K. slovaca
87, 96, 97, 316, 317, 456, 464, 466
K. arvensis
K. pseudolongifolia
K. serpentinicola
80, 81, 83, 372, 419, 454-456, 465, 467
K. norica
6, 50, 51, 93
K. carinthiaca
15
Carinthiaca & North Arvensis Group
K. involucrata
453
K. montana
451, 452
Montana Group
K. dipsacifolia
84, 85, 342, 422, 463
Alpine Dipsacifolia Group
K. dipsacifolia
K. subcanescens
370, 375, 377, 378
K. lebrunii
404, 408
K. arvernensis
K. foreziensis
379-383, 385, 387, 388
K. basaltica
K. godetii
386, 389, 460-462
SW European Group
0.1

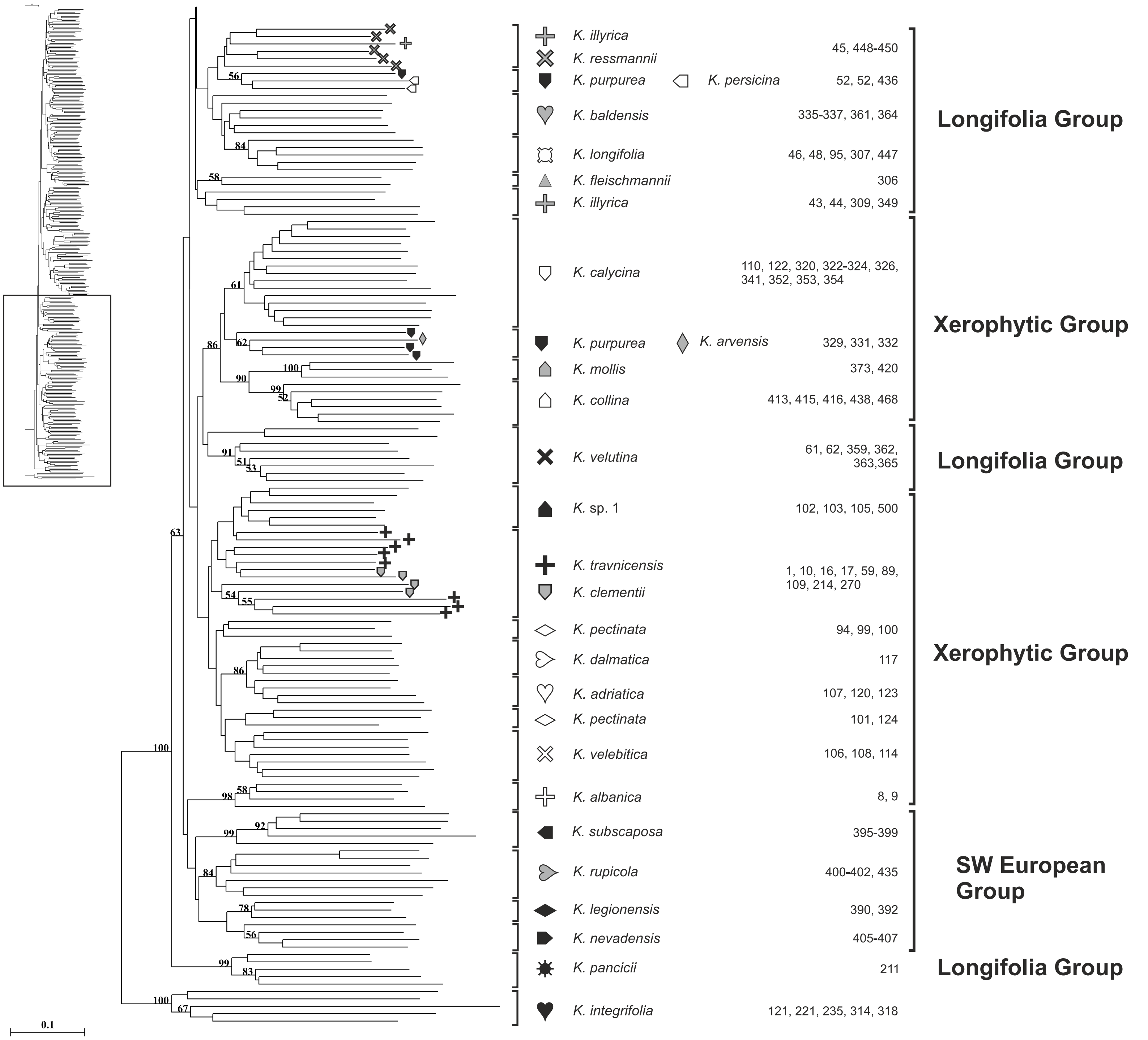

Additional file 6: Figure S5. Relationships among populations of 51 species of *Knautia* sect. *Trichera* visualised by a Neighbour-joining tree based on Nei-Li distances derived from AFLP data. The tree is rooted with *K. integrifolia* from sect. *Tricheroides.* Bootstrap support values (based on 1000 replicates) above 50% are given for branches with ≥ 3 terminals. Symbols for the species correspond to those used in Fig. 1. Separate labeling at the tips of branches was used when individuals of different taxa were intermixed. Population numbers given to the right of the species' names correspond to Supplementary Table S1. Species groups given at the right margin are detailed in Table 2 and the Additional File 8.
