# Supplementary material for: Patterns of rapid diversification in heteroploid Knautia sect. Trichera (Caprifoliaceae, Dipsacoideae), one of the most intricate taxa of the European flora
Source: BMC Evol Biol. 2016 Oct 10;16:204. doi: 10.1186/s12862-016-0773-2 (PMC5057222; doi:10.1186/s12862-016-0773-2)

Additional file 7: Figure S6. Amplified Fragment Length Polymorphism (AFLP) variation in 251 populations of 51 species of *Knautia* sect. *Trichera* visualised as NeighbourNet diagram based on uncorrected P distances. The colours of individual branches indicate ten genetic clusters as identified by K-means clustering. This is an enlargeable version of Fig. 6 with labelling of terminal splits with species names and population IDs.

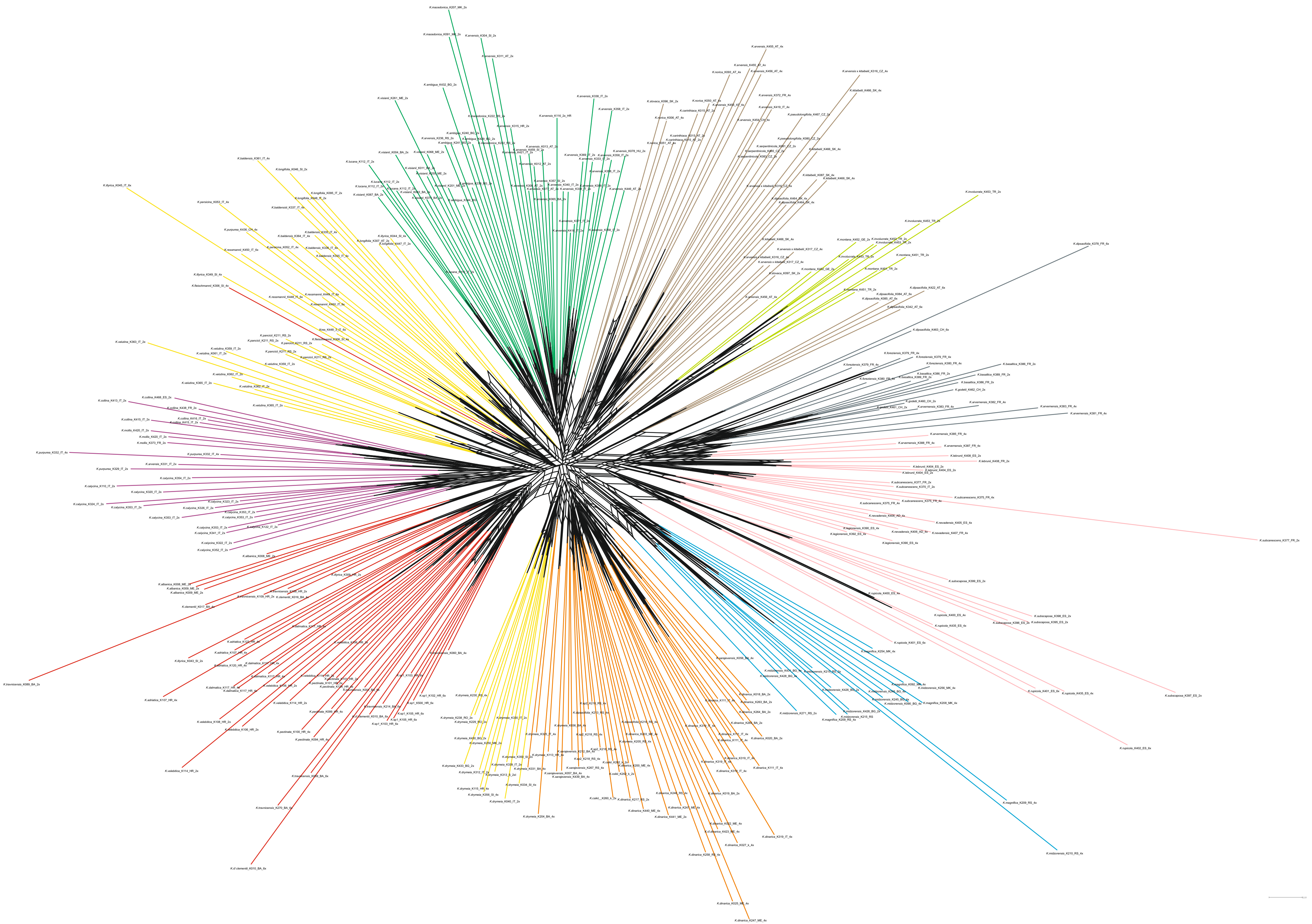

Supplement: Additional file 7: Figure S6. — Amplified Fragment Length Polymorphism (AFLP) variation in 251 populations of 51 species of Knautia sect. Trichera visualised as NeighbourNet diagram based on uncorrected P distances. The colours of individual branches indicate ten genetic clusters as identified by K-means clustering. This is an enlargeable version of Fig. 6 with labelling of terminal splits with species names and population IDs (PDF 1493 kb) [file 12862_2016_773_MOESM7_ESM.pdf]
