# Supplementary material for: Patterns of rapid diversification in heteroploid Knautia sect. Trichera (Caprifoliaceae, Dipsacoideae), one of the most intricate taxa of the European flora
Source: BMC Evol Biol. 2016 Oct 10;16:204. doi: 10.1186/s12862-016-0773-2 (PMC5057222; doi:10.1186/s12862-016-0773-2)

Additional file S8:

Patterns of rapid diversification in heteroploid *Knautia* sect. *Trichera* (Caprifoliaceae, Dipsacoideae), one of the most intricate taxa of the European flora

Božo Frajman[1*], Ivana Rešetnik[2*], Marjan Niketić[3], Friedrich Ehrendorfer[4**] & Peter Schönswetter[1]

[*]Contributed equally to this work
[**]Corresponding author

[1]Institute of Botany, University of Innsbruck, Sternwartestraße 15, A-6020 Innsbruck, Austria. [2]Faculty of Science, University of Zagreb, Marulićev trg 20, HR-10000 Zagreb, Croatia. [3]Natural History Museum, Njegoševa 51, 11000 Belgrade, Serbia. [4]Department of Botany and Biodiversity Research, University of Vienna, Rennweg 14, A-1030 Vienna, Austria.

Informal classification of diploid and polyploid *Knautia*

Only two groups previously established for diploids [24] remain unchanged in the heteroploid data set. The geographically disjunct **Montana Group** includes *K. involucrata* and *K. montana* distributed from Anatolia eastwards. The widely distributed **South Arvensis Group** spans from the Alps south- and southeastwards and comprises the flower-colour-differentiated but genetically inseparable [24] *K. ambigua* and *K. macedonica*, the monocarpic *K. visianii* and *K. lucana* as well as accessions of *K. arvensis* from the south of its distribution area. The two latter groups form separate clusters in the K-means analysis of the AFLP data (Fig. 6 in the main text).

The **Midzorensis Group** includes *K. midzorensis* and *K. magnifica* from the southern and eastern Balkans. Whereas *K. midzorensis* possesses diploid and tetraploid cytotypes, *K. magnifica* is exclusively tetraploid [25]; however, separation of the two species appears highly questionable (Additional file 6: Figure S5, Additional file 8: Figure S9). This relatively well-supported group (BS 61, Additional file 6: Figure S5; BS 77, Additional file 8: Figure S9), which also constitutes a separate K-means cluster (Fig. 6), possesses only haplotypes of the Red Haplotype Group (Additional file 8: Figure S9); its ribotypes share splits with members of several other groups (Fig. 3).

The inclusion of polyploids renders the Dinarica and Drymeia Groups, previously delimited on the basis of diploids [24], genetically inseparable (Fig. 6). Therefore, they are here combined in the **Drymeia & Dinarica Group**. Alongside the heteroploid (diploid and tetraploid) *K. csikii*, *K. dinarica* and *K. drymeia*, this joint group also includes the tetraploid Balkan endemics *K. sarajevensis* and *K. dipsacifolia* subsp. *lancifolia*. This arrangement (excluding *K. subcanescens* falling into the SW European Group) corresponds to Szabó's [105] subsection *Purpurascentes*. According to the regional datasets, central European accessions of *K. drymeia* form a loose cluster (Additional file 8: Figure S8), whereas three unsupported subgroups emerge on the Balkan and Apennine Peninsulas (Additional file 8: Figure S9): The first subgroup corresponds to the Dinarica Group and covers di- and tetraploid *K. dinarica* from the Balkans, tetraploid *K. dinarica* subsp. *silana* from southern Italy as well as two populations of the Balkan endemic *K. sarajevensis*. The second subgroup is an assemblage of di- and tetraploid *K. dinarica*, as well as of tetraploid *K. dipsacifolia* subsp. *lancifolia* (K213 and K216), *K. drymeia*, *K. sarajevensis* and an unidentifiable tetraploid population (*K.* sp. 2; K218) from Serbia. The third subgroup corresponds to the Drymeia Group and encompasses all diploid and a few tetraploid

accessions of *K. drymeia* including a tetraploid population from the central Apennines (see Rešetnik et al. [95] for a detailed analysis of *K. drymeia*) as well as diploid *K. csikii*. The K-means clustering of AFLP data unites the majority of the *K. drymeia* samples with the Longifolia and Pancicii Groups, whereas the remaining samples form a separate cluster (Fig. 6). The heterogeneity of the Drymeia and Dinarica Group is also evident in the ITS NeighbourNet (Additional file 4: Figure S3), where accessions are scattered in different parts of the network. Accessions of *K. dinarica* intermingle with the South Arvensis Group and the North Arvensis Groups whereas *K. drymeia* displays relationships with the latter two groups, the Xerophytic Group and the SW European Group.

The **Xerophytic Group** can be divided into three geographically correlated subgroups. The strongly supported (BS 98, Additional file 6: Figure S5) first group is constituted by the diploid Dinaric *K. albanica*, the second is restricted to the northwestern Balkan Peninsula and includes tetraploid *K. adriatica* and *K. dalmatica*, di- and tetraploid *K. pectinata*, tetra- and hexaploid *K. clementii*, di, tetra- and hexaploid *K. travnicensis*, diploid *K. velebitica* and an unidentifiable hexaploid entity from Velebit (*K.* sp. 1). Several species do not form tight clusters whereas the divergence of the sister species pair *K. adriatica* and *K. dalmatica* is strongly supported (BS 86, Additional file 6: Figure S5; BS 93, Additional file 8: Figure S9). The third, strongly supported subgroup (BS 86, Additional file 6: Figure S5), which forms a separate K-means cluster in the AFLP analysis (Fig. 6), extends from the Pyrenees to Sicily. It comprises *K. calycina* from the central Apennines and Sicily, the sister species (BS 90; Additional file 6: Figure S5) *K. mollis* from the southwestern Alps and *K. collina* distributed from the southwestern Alps to the Pyrenees, as well as accessions of *K. arvensis* and *K. purpurea* from the northern Apennines. As in our previous study [24], the intergrading sympatric *K. collina* and *K. mollis* are strongly supported sisters (Additional file 6: Figure S5, Additional file 8: Figure S9) while other interspecific relations remain elusive including the possible connection of *K. arvensis* and *K. purpurea* from the northern Apennines to *K. calycina* recovered only within one regional AFLP dataset (BS 62, Additional file 8: Figure S9). Our previous study based on diploid accessions included in the Xerophytic Group also the diploid *K. velutina* from the southern Alps [24]. However, K-means clustering applied to the heteroploid data set (Fig. 6) rather assigned it to the Longifolia Group, where it is also included in the regional data set (Additional file 8: Figure S8). The close relationships of the Xerophytic Group with members of Longifolia and Drymeia Groups based on ITS data is indicated with several shared splits (Additional file 4: Figure S3). The evident heterogeneity of the Xerophytic Group is supported by the presence of five of the totally six haplotype groups (Additional file 8: Figure S9).

The previously identified, strongly supported monospecific **Longifolia Group** [24] is strongly inflated with the inclusion of polyploids and comprises also tetraploid *K. baldensis*, *K. fleischmannii*, and *K. persicina*, hexaploid *K. ressmannii*, di-, tetra- and hexaploid *K. illyrica* (Fig. 6) as well as diploid *K. velutina*, a single diploid accession of *K. arvensis* (population 331) and di- and tetraploid *K. purpurea*. Diploid accessions of the four before-mentioned entities were previously included in the Xerophytic Group [24]. Most prominently, the Serbian endemic *K. pancicii* previously segregated in the monospecific Pancicii Group [24] is deeply nested in the Longifolia Group upon the inclusion of polyploid accessions. Alongside the Longifolia Group K-means clustering includes also a coherent part of the Drymeia and Dinarica Group in the same cluster (Fig. 6). Four different haplotype groups were retrieved in the Longifolia Group (Additional file 8: Figure S8). The groups' pronounced heterogeneity is also evident in the ITS data, where diploid and polyploid taxa share splits with various lineages from several major assemblages (Fig. 3, Additional file 4: Figure S3).

The **SW European Group**, which is constituted by two K-means clusters, one of which is exclusive and one is shared with the hexaploid Dipsacifolia Group (Fig. 6), comprises several lineages forming two unsupported, unrelated groups in the AFLP Neighbour-joining tree (Additional file 6: Figure S5). The first, strictly Iberian, group includes diploid *K. subscaposa* together with tetraploid *K. legionensis* and *K. nevadensis* as well as tetra- and hexaploid *K. rupicola*, which form mostly supported monospecific clades (Additional file 6: Figure S5, Additional file 8: Figure S7). All four species are characterised by significantly larger monoploid genome sizes than found in any other species of sect. *Trichera* in spite of identical base chromosome base numbers [25]. The larger genome size is likely connected with the amplification of certain classes of repetitive DNA, tandem repeats or transposable elements [25] and provides additional evidence of their close relationship despite their different morphology (e.g., low-growing *K. rupicola* and *K. subscaposa* with strongly dissected leaves vs. tall-

growing *K. nevadensis* with usually entire leaves). However, interspecific relationships were not resolved and the four species did not group in the regional AFLP dataset (Additional file 8: Figure S7). The second group includes diploid and tetraploid *K. subcanescens* from the western Alps, the highly supported (BS 99, Additional file 6: Figure S5) diploid *K. lebrunii* from the eastern Pyrenees, as well as diploid *K. basaltica* and tetraploid *K. arvernensis* and *K. foreziensis* from the French Massif Central and diploid *K. godetii* from the Swiss Jura and the Massif Central. All species with the exception of *K. lebrunii* fail to form monophyletic lineages (Additional file 6: Figure S5) and identification was far from straight-forward (PS & BF, field obs.). The regional AFLP dataset (Additional file 8: Figure S7) indicates the heterogeneity of *K. arvernensis*, which clusters with various other species, which might be indicative of recurrent formation of tetraploid *K. arvernensis*. The ITS data, in contrast, rather support close relationships among the constituents of the SW European Groups as all ribotypes (except *K. arvernensis* K382) are grouping in a small section of the network (Fig. 3).

On the diploid level the *K. arvensis* complex was divided into two clearly separated groups [24], that is (1) populations of *K. arvensis* from the Alps southwards together with *K. ambigua*, *K. lucana*, *K. macedonica* and *K. visianii* forming the **South Arvensis Group** characterised in the main text and (2) populations of *K. slovaca*, *K. pseudolongifolia* and *K. serpentinicola* (the latter two were only recently described by Kolář et al. [40] and thus referred to as *K. arvensis* in our previous study) from north of the Alps, forming the North Arvensis Group. This group and the previously [24] separated Carinthiaca Group (comprising only the Eastern Alpine endemic *K. carinthiaca*, known from a single population [91]), are inseparable upon the addition of polyploids. Alongside the before-mentioned diploids, the resultant **Carinthiaca & North Arvensis Group** comprises tetraploid *K. arvensis* populations from the western and north-eastern margin of the Alps, *K. kitaibelii*, *K. arvensis* × *K. kitaibelii*, and *K. dipsacifolia* from Slovakia as well as *K. norica*. In the AFLP NeighbourNet (Fig. 6) and the Neighbour-joining tree (Additional file 6: Figure S5) this group is tightly clustering or monophyletic, albeit unsupported, whereas in the regional AFLP Neighbour Net it separates into two groups (Additional file 8: Figure S8). The heterogeneity of this group is supported by ITS data with accessions distributed across much of the NeighbourNet (Additional file 4: Figure S3).

Finally, hexaploid populations of *K. dipsacifolia* from the Alps (Fig. 6) are separated both from the tetraploid Balkan populations of that species pertaining to the Drymeia & Dinarica Group and the central European populations falling into the Carinthiaca & North Arvensis Group and form the **Alpine Dipsacifolia Group**.

References not cited in the main text

105. Szabó Z. A *Knautia* génusz monographiája. Kiadja a Magyar tudományos academia. Budapest; 1911.

Figure S7. NeighbourNet diagram visualising genetic relationships among accessions of *Knautia* sect. *Trichera* from southwestern Europe (Iberian Peninsula to Western Alps; for the geographic delimitation see Fig. 2B) based on Amplified Fragment Length Polymorphism (AFLP). Terminal splits are coded according to ploidy level: black, diploid; red, tetraploid; green, hexaploid. Numbers along the splits are bootstrap values > 50% derived from a neighbour-joining analysis; they are given for major groups only. Coloured dots at the tips of the branches correspond to the groups of plastid DNA haplotypes shown in Fig. 2. Symbols for species correspond to Fig. 1; tightly clustering multiple accessions of a species are shaded in grey and labelled with the species name. Genetic groups as defined in Table 2 are indicated by colour shading. For each species typical leaf shapes are provided in order to enable comparison of genetic groups with this taxonomically important character.

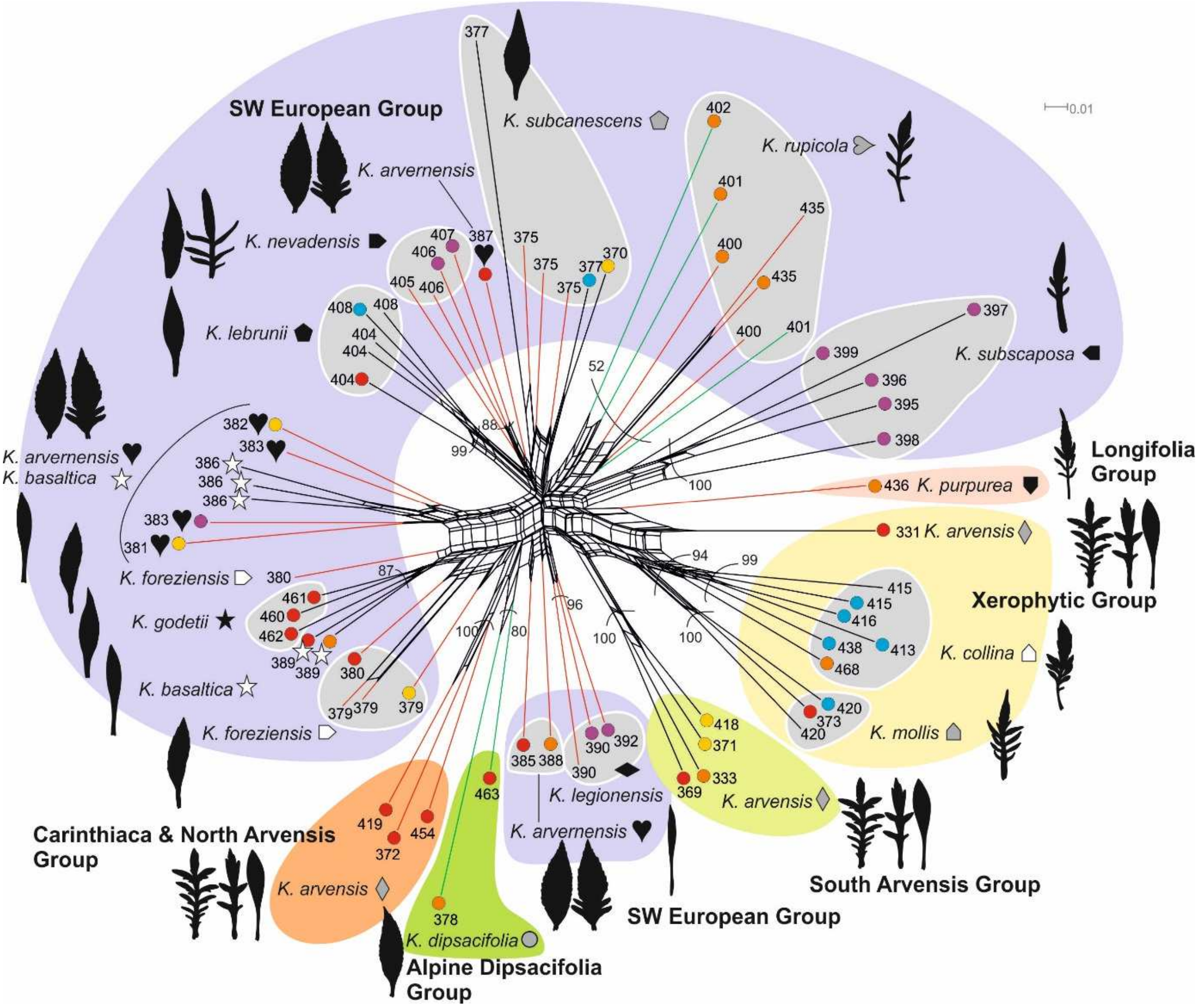

Figure S8. NeighbourNet diagram visualising genetic relationships among accessions of *Knautia* sect. *Trichera* from central Europe (Eastern Alps and north-easterly adjacent areas, northern-most Balkan Peninsula; for the geographic delimitation see Fig. 2B) based on Amplified Fragment Length Polymorphism (AFLP). Terminal splits are coded according to ploidy level: black, diploid; red, tetraploid; green, hexaploid. Numbers along the splits are bootstrap values > 50% derived from a neighbour-joining analysis; they are given for major groups only. Coloured dots at the tips of the branches correspond to the groups of plastid DNA haplotypes shown in Fig. 2. Symbols for species correspond to Fig. 1; tightly clustering multiple accessions of a species are shaded in grey and labelled with the species name. Genetic groups as defined in Table 2 are indicated by colour shading. For each species typical leaf shapes are provided in order to enable comparison of genetic groups with this taxonomically important character.

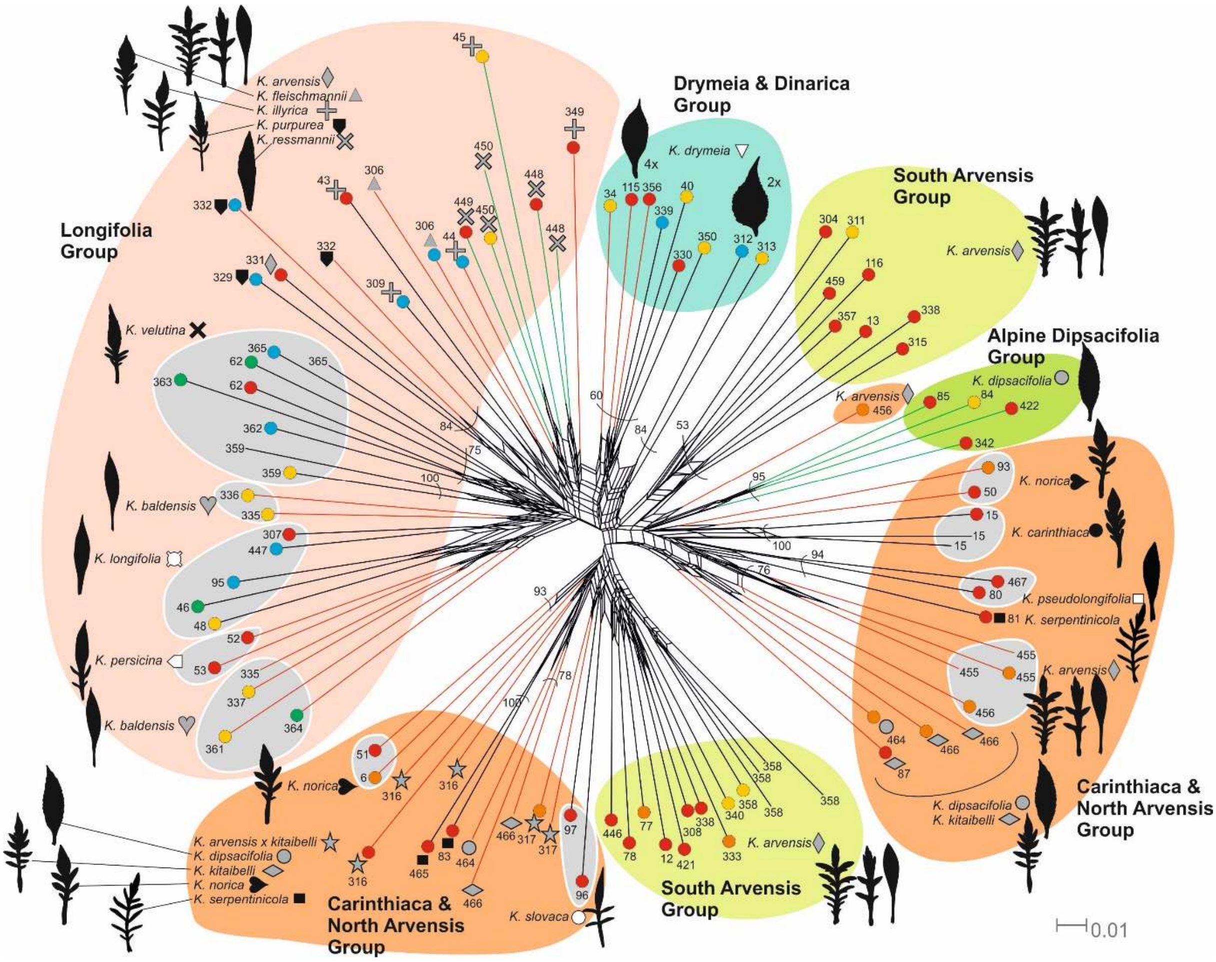

Figure S9. NeighbourNet diagram visualising genetic relationships among accessions of *Knautia* sect. *Trichera* from the central and southern Apennine Peninsula, the Balkan Peninsula – except for its northern-most part – and Western Asia (for the geographic delimitation see Fig. 2B) based on Amplified Fragment Length Polymorphism (AFLP) are coded according to ploidy level: black, diploid; red, tetraploid; green, hexaploid. Numbers along the splits are bootstrap values > 50% derived from a neighbour-joining analysis; they are given for major groups only. Coloured dots at the tips of the branches correspond to the groups of plastid DNA haplotypes shown in Fig. 2. Symbols for species correspond to Fig. 1; tightly clustering multiple accessions of a species are shaded in grey and labelled with the species name. Genetic groups as defined in Table 2 are indicated by colour shading. For each species typical leaf shapes are provided in order to enable comparison of genetic groups with this taxonomically important character.

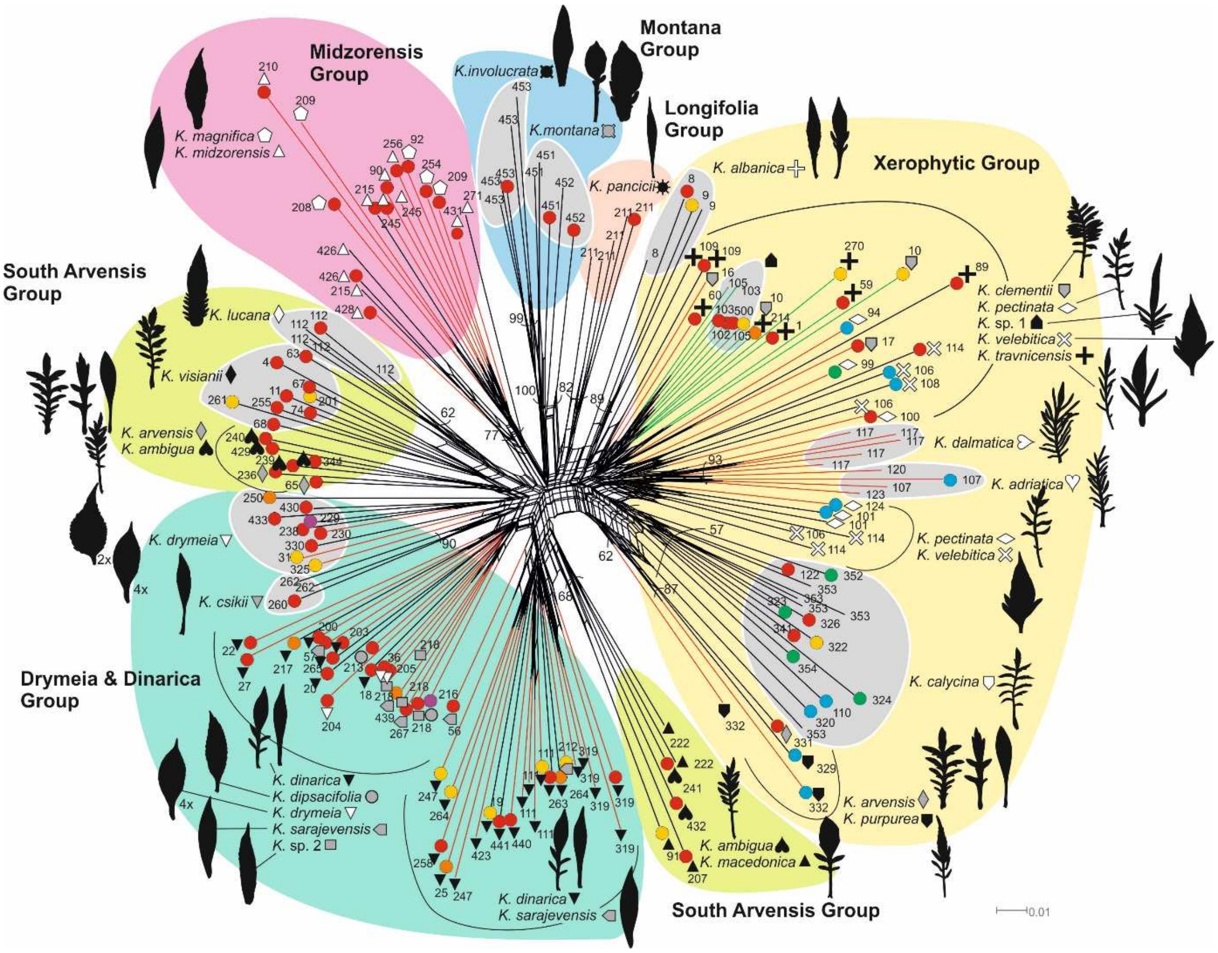

Supplement: Additional file 8: — Informal classification of diploid and polyploid Knautia (containing Figures S7–S9) (PDF 664 kb) [file 12862_2016_773_MOESM8_ESM.pdf]
